# Conformational switching modulates excited-state pathways in a cofacial perylene dimer

**DOI:** 10.1039/d5sc09512c

**Published:** 2026-01-22

**Authors:** Giovanni Bressan, Denis Hartmann, Jonathan Brouwer, Erico M. Braun, James N. Bull, Timothy A. Barendt

**Affiliations:** a School of Chemistry, University of East Anglia Norwich NR4 7TJ UK g.bressan@uea.ac.uk; b School of Chemistry, University of Birmingham Birmingham B15 2TT UK t.a.barendt@bham.ac.uk; c Instituto de Fisica, Universidade Federal do Rio Grande do Sul Av. Bento Gonçalves Porto Alegre 9500 Brazil

## Abstract

Controlling excited-state pathways in supramolecular chromophore assemblies is key to designing next-generation optoelectronic and photonic materials. Here we elucidate the conformation-dependent photophysics of a flexible perylene diimide (PDI) dimer, valPDI_2_, which undergoes reversible solvent-driven switching between two distinct dimer geometries, within the same structure. Exciton-coupling calculations and ultrafast spectroscopy show that in chloroform the dimer adopts an open, weakly coupled geometry that supports slow, partial excimer formation due to structural inhomogeneity within the excited state potential. In contrast, in polar DMSO/water the dimer collapses into a cofacial stacked conformer that enables barrierless, sub-200 femtosecond excimer formation, a subset of which forms a multiexciton state over tens of picoseconds. Half-broadband 2D electronic spectroscopy reveals conformation-dependent vibrational coherences, with nuclear wavepacket motion along the π-stacking coordinate promoting vibrationally coherent excimer formation in the closed conformer. These findings demonstrate that environmentally driven conformational control offers a powerful non-covalent strategy to modulate excimer and multiexciton dynamics in PDI assemblies. More broadly, they establish supramolecular switching as a general design principle for tuning excited-state behaviour in flexible organic chromophore arrays, with implications for the development of responsive optoelectronic and energy-conversion materials.

## Abstract 2

Ultrafast coherent spectroscopies uncover conformation-dependent excimer dynamics in a switchable bis-perylene diimide macrocycle, behaviour that is key to modulating excited state pathways in optoelectronic materials.

## Graphical abstract

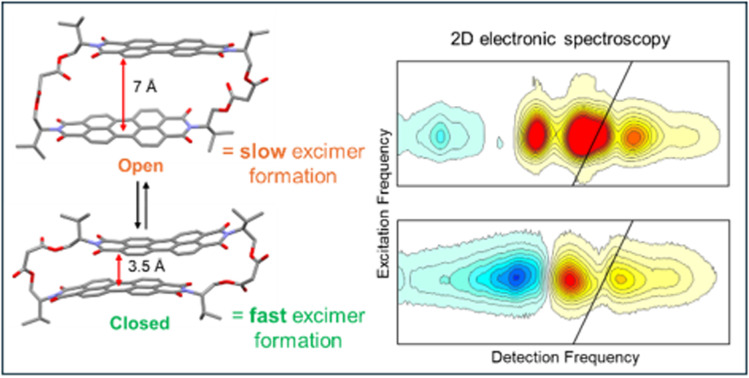

## Introduction

Excited-state interactions in multichromophore assemblies lie at the heart of many optoelectronic processes, from energy transport to charge separation and luminescence. Among these interactions, excimer formation in π-stacked systems has attracted considerable interest since its first observation in concentrated pyrene solutions 70 years ago.^1^ Excimers (EX) represent adiabatic mixtures of singlet excitons ^1^(S_1_S_0_)-also known as local excited (LE)- and charge-transfer (CT) wavefunctions and can either act as traps or facilitate useful processes such as charge separation and multiexciton (ME) generation *via* singlet fission (SF).^2–10^ Supramolecular architectures based on the perylene diimide (PDI) building block provide an ideal platform for probing these phenomena, combining strong absorption in the green region of the visible spectrum, high photostability, and structural tunability *via* functionalisation at the imide or bay positions.^11–14^ Upon photoexcitation, rylene dimers access mixed electronic states with EX, CT, and correlated triplet-pair ME character, whose balance and time evolution are strong function of relative chromophore orientation, distance and environment and ultimately dictate the dimer's photophysics.^4,7,15–23^

Therefore, understanding how geometry and environment jointly govern the mixed state behaviour is of central importance to the development of photoactive organic materials. Further, coherently excited nuclear wavepackets have been shown to drive sub-picosecond relaxation out of the Franck–Condon region, suggesting the possibility of harnessing vibrational coherence to control the fate of mixed excited states.^4,16,17,19,24,25^

However, mixed state character and dynamics are difficult to control within a single dimer structure. Typical tuning strategies rely on covalent modifications, which may alter the electronic structure, and require the synthesis of a series of dimer structures to systematically vary interchromophore distance and/or orientation. An alternative strategy leverages non-covalent stimuli, allowing a single dimer structure to explore multiple conformations and thus excitonic coupling regimes and excited state evolution in distinct dimer geometries. A notable example of such behaviour was recently reported by Thakur *et al.*,^26^ who demonstrated solvent polarity-controlled switching between open and folded conformations of a flexible PDI dimer, accompanied by drastic changes in charge separation dynamics. Despite their relevance to solid-state devices, the photophysics of chromophore dimers capable of reversibly switching between weak (or null) and strong excitonic couplings remain scarcely explored, motivating the present study.

**Scheme 1:**
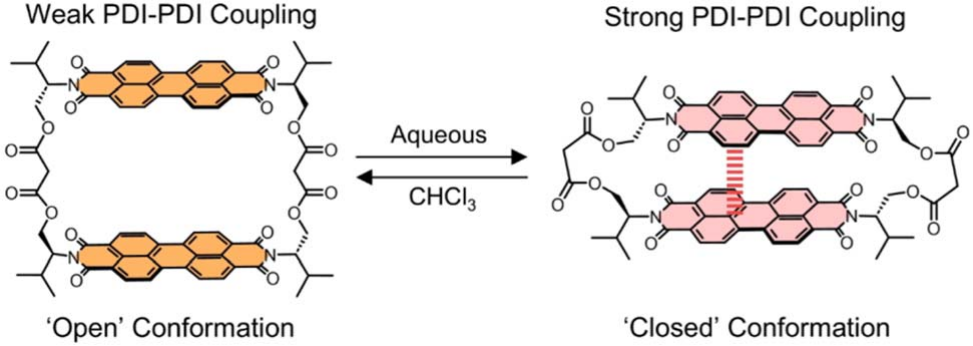
Molecular structure and solvent-dependent conformers of valPDI_2_.

Here, we investigate the conformation dependent photophysics and ultrafast (coherent) dynamics of valPDI_2_, a switchable and discrete PDI dimer recently reported by some of us, whose structure is shown in Scheme 1.^27^ This macrocycle incorporates a flexible L-valinol-malonate ester linker to connect two PDIs *via* their imide N positions which, in contrast to our previous perylene core-connected PDI dimer,^24,28^ affords valPDI_2_ with molecular recognition properties. This includes interactions with solvent molecules, generating distinct solvent-dependent conformations of valPDI_2_. In moderately polar CHCl_3_ (*ε*_r_ = 4.81), the discrete PDI dimer adopts an “open” geometry supporting weak excitonic coupling; in polar, protic 1 : 1 DMSO/water (*ε*_r_ = 66),^29^valPDI_2_ collapses into a cofacial, strongly coupled “closed” conformation due to PDI hydrophobicity. Such solvent-controlled switching enables reversible modulation of the excitonic coupling and associated excited-state dynamics without the need for alterations of the covalent structure.

Our study combines fs transient absorption (TA) and half-broadband two-dimensional electronic spectroscopy (HB2DES), supported by quantum chemical modelling. Density functional theory (DFT) and extended tight-binding (XTB)^30^ calculations confirm the presence of solvent-dependent conformers, which populate EX and, in DMSO/water, ME states over markedly different timescales. In CHCl_3_, EX formation occurs over several ps following photoexcitation *via* LE (*i.e.* the Franck–Condon region of the S_1_ potential energy surface, PES) state branching. This arises from multiple dimer conformers in thermal equilibrium within the S_0_ PES. In contrast, in DMSO/water, the preorganised collapsed cofacial geometry enables near-barrierless and sub-200 fs EX formation, a non-negligible fraction of which evolves into a ME correlated triplet pair state over tens of ps, in parallel with charge separation and recombination repopulating S_0_. This indicates that solvent polarity, intermolecular H-bonding and conformational switching can accelerate EX formation by more than two orders of magnitude and gate ME generation and charge separation. Further, 2DES beatmap analysis suggests that the sub-200 fs excimer formation is driven by coherent nuclear motion along the PDI–PDI π–π coordinate, aiding population transfer from the LE to the EX regions of the excited state PES.

Together, our findings show that conformational switching, medium polarity, H-bond networks and nuclear wavepackets collectively modulate EX and ME state generation in flexible PDI dimers in solution, offering new design principles for organic materials with tuneable excited-state properties. Moreover, our results indicate that the supramolecular arrangement -and, consequently, the optical response- of PDI-based systems in solid-state devices could be controlled by the dielectric constant and the protic nature of the surrounding polymer matrix. This environmentally driven, reversible control mechanism is a novel tool for tuning excited-state behaviour *via* conformational regulation, rather than synthetic redesign.

## Results and discussion

### Steady-state spectroscopy, molecular structure and excitonic coupling

**Fig. 1:**
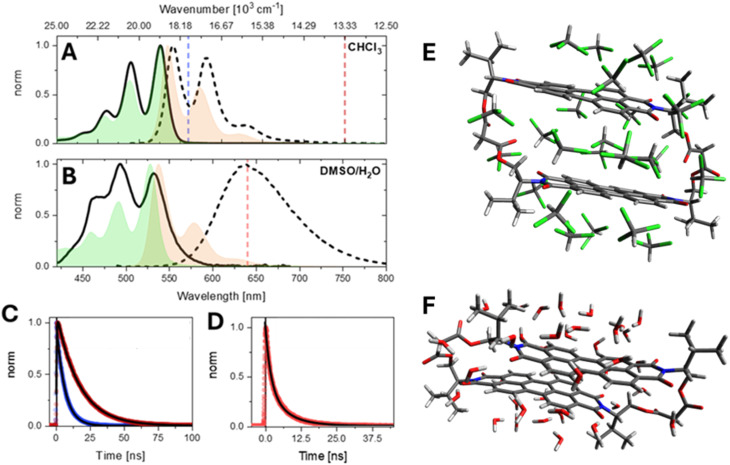
Solvent-dependent spectroscopy and conformers of valPDI_2_. (A) Normalised steady-state absorption (solid) and emission (dashed) spectra of valPDI_2_ and the reference monomer, refPDI (absorption in green and emission in orange). Fluorescence spectra were excited at *ν̃*_Exc_ = 21 000 cm^−1^ (478 nm). (B) Same as (A) for 1 : 1 DMSO/water. (C) ns fluorescence emission traces of valPDI_2_ in CHCl_3_ measured at 18 000 cm^−1^ (blue) and 13 500 cm^−1^ (dark red). Emission wavelength marked also with dash line in (A) and (B). (D) Same as (C) for valPDI_2_ in 1 : 1 DMSO/water at 15 700 cm^−1^; single/multiexponential fits (fitted time constants given in text) are shown as black solid lines in (C) and (D). All traces were recorded after photoexcitation at 21 000 cm^−1^. (E and F) Molecular structures of the valPDI_2_ dimer with the proposed “open” (0.7 nm PDI–PDI separation) and “closed” (0.35 nm PDI–PDI separation) macrocycle conformations in CHCl_3_ and DMSO/water. Both conformers have been obtained by optimisation of XRD structures^27^ with 25 explicit CHCl_3_ or 40 explicit H_2_O solvent molecules using XTB. XTB trajectories out to 200 ps at 300 K show that the cavity in the open geometry (E) supports five or six CHCl_3_ molecules. In the closed geometry shown in (F), the PDI units remain closely π-stacked with no solvent intercalation.

The normalised steady-state electronic absorption and emission spectra of valPDI_2_ in CHCl_3_ and 1 : 1 DMSO/water are reported in Fig. 1A and B. For comparison, the absorption and emission spectra of the reference monomer (refPDI, structure shown in SI, Fig. S1) in the same solvents are overlaid.

In CHCl_3_, valPDI_2_ (1A, solid) shows an absorption maximum at 526 nm (19 000 cm^−1^) with a well-resolved vibronic progression (≈1350 cm^−1^ spacing), attributed to Raman-active C=C stretches displaced along the S_1_ ← S_0_ transition, as typically encountered in polycyclic aromatic hydrocarbons (PAHs).^14,31^ This spectrum closely resembles that of the monomeric refPDI (green shade), though valPDI_2_ displays a more intense 0–1 vibronic peak at 492 nm (20 360 cm^−1^) relative to the 0–0. Its fluorescence (1A, dashed; fluorescence quantum yield (FQY) = 0.91) upon 478 nm excitation has a quasi-mirror image relation to the absorption but is slightly broadened and redshifted by ≈300 cm^−1^ compared to refPDI. A weak emission tail extending to 13 300 cm^−1^ – absent in refPDI*–* is observed. Nanosecond time-resolved fluorescence (478 nm excitation, 1C) reveals wavelength-dependent lifetimes: 6 ns at 570 nm (blue, 17 540 cm^−1^) and 17 ns at 750 nm (dark red, 13 330 cm^−1^), the latter has a ≈0.5 ns rise time. This suggests multiple emissive states or an excited-state equilibrium for valPDI_2_ in CHCl_3_. Conversely, in CHCl_3_refPDI exhibits a wavelength independent 5 ns lifetime (SI, Fig. S2).

In a polar 1 : 1 DMSO/water solvent mixture (1B, solid), the absorption of valPDI_2_ broadens and redshifts (onset at *ca* 600 nm), with an inversion of the vibronic peak intensities (0–0 at 533 nm, 0–1 at 494 nm). Similar behaviour was reported by Cadena *et al.* upon addition of increasing amounts of water to a DMF solution of a covalently tethered PDI oligomer.^32^ In DMSO/water, fluorescence (1B, dashed) is weaker (FQY = 0.065), redshifted (*λ*_max_ = 640 nm), lacks vibronic structure, and exhibits a biexponential fluorescence decay (1.9 and 6.7 ns; weighted average = 3 ns, 1D) independent of emission wavelength. By comparison, refPDI in DMSO/water has a 1.5 ns fluorescence lifetime (SI, Fig. S2). This is expected, as water quenches photoexcited dyes *via* electronic-vibrational excitation energy transfer.^33^ The hypothesis of such quenching being due to PDI aggregation in polar media is ruled out by the strong similarity between the absorption spectra of refPDI in CHCl_3_ and DMSO/water. The ≈10-fold decrease in FQY of valPDI_2_ in DMSO/water (*vs.* in CHCl_3_) and their comparable lifetimes indicate a reduced emission transition dipole moment (TDM) in polar solvents. Weak, broad and redshifted emission is commonly observed for PDI excimers, whose role in mediating or hindering ME generation has been debated over the past decade.^4,7,9,10,34^ We also note that, despite ME states being dark, they can gain oscillator strength *via* Herzberg–Teller coupling to energetically close bright states (such as S_1_). Intensity borrowing leads to redshifted emission, strongly resembling EX features.^34^

The pronounced solvent dependence of the valPDI_2_ photophysics can be attributed to solvent-induced conformational changes of the discrete macrocycle dimer structure. In CDCl_3_, ^1^H NMR spectroscopy indicates the presence of an open conformation where the flexible ligands expand to accommodate solvent between the PDI units.^27^ This assignment is supported by XTB calculations, in which the crystal structure of the host–guest valPDI_2_-coronene cocrystal^27^ (with the coronene guest removed) was optimised using the SOLVATOR method with 25 explicit CHCl_3_ solvent molecules, as implemented in ORCA 6.1.0,^35^ revealing solvation of the interchromophoric cavity by 5–6 CHCl_3_ molecules, resulting in an estimated PDI–PDI separation of approximately 0.7 nm (see Fig. 1E). For PDIs, chlorinated solvents preferentially stabilise solute–solvent interactions, hence inhibiting the π–π stacking solute–solute interactions that are typically responsible for the formation of dimers or extended aggregates.^36^

In DMSO/water, the valPDI_2_ macrocycle adopts a closed collapsed conformer geometry (shown in Fig. 1F), driven by intramolecular π–π stacking between the two PDIs since, in this medium, these interactions dominate over solute-(polar) solvent interactions. This conformer was characterised by 1D and 2D ^1^H NMR spectroscopy in solution.^27^ Importantly, the addition of increasing amounts of water to DMSO does not drive a continuous evolution of the valPDI_2_ geometry. Instead, isosbestic points in the absorption/circular dichroism solvent–solvent titrations^27^ indicate a two-state equilibrium, *i.e.* switching between “open” and “closed” conformations, rather than a gradual evolution through intermediate conformers with multiple equilibria.

Further support for this conformer structure was obtained by optimising the crystal structure^27^ of valPDI_2_ in SOLVATOR with 40 explicit water molecules using the XTB method. While the SOLVATOR framework requires neat water, this is an adequate approximation to the 1 : 1 DMSO/water solvent used, since intramolecular π–π aggregation is observed for valPDI_2_ in both 1 : 1 DMSO/water and water with only 0.1% DMSO. Significantly, the calculated structure does not show any water molecules intercalated between the PDI chromophores. This conformer displays a reduced PDI–PDI separation, ≈0.35 nm, accompanied by a longitudinal slip along the PDI long axis (*i.e.*, aligned with the PDI S_1_ ← S_0_ TDM).

Excitonic coupling strength (*J*_Tot_) was estimated using Spano's method,^37,38^ which relates the 0–1/0–0 vibronic peak area ratio *R*_0–1_*/R*_0–0_ to excitonic coupling magnitude and sign. In CHCl_3_, *R*_0–1_/*R*_0–0_ = 0.8 (*vs.* 0.68 for refPDI); in DMSO/water, *R*_0–1_/*R*_0–0_ = 1.13, indicating positive (H-type) excitonic interactions in both solvents. Peak area ratios were extracted by fitting absorption spectra to three Gaussian components (see SI, Fig. S3). For a vibronic spacing of 1350 cm^−1^, the determined *R*_0–1_/*R*_0–0_ values yield total excitonic coupling strengths *J*_Tot_ of +210 cm^−1^ in CHCl_3_ and +473 cm^−1^ in DMSO/water (details of the calculation are reported in the SI). These results indicate that valPDI_2_ behaves as a weakly and strongly coupled H-dimer in CHCl_3_ and in DMSO/water, respectively (Scheme 1), consistent with the proposed and calculated open and closed conformers populated in the two solvent media (Fig. 1E and F).

Following Spano's work,^39^ the total excitonic coupling *J*_Tot_ is dictated by the interference between long-range coulombic *J*_Coul_ and short-range charge-transfer *J*_CT_ terms:

1 *J*_Tot_ = *J*_Coul_ + *J*_CT_

Whilst *J*_Coul_ can be approximated using the point-dipole model,^40^ where the coupling scales as the inverse cube of the distance between TDMs, this model tends to overestimate coupling strengths at chromophore separations comparable to molecular dimensions. For a more reliable estimate, we used the transition electrostatic potential (trESP) method, as implemented in Multiwfn, with TDMs obtained from time-dependent (TD)-DFT (Gaussian 16.B01).^24,41–43^

The trESP method (details given in the SI) yielded *J*_Coul_ values of +261 cm^−1^ and +462 cm^−1^ for the open and closed valPDI_2_ conformers, respectively, aligning well with Spano's fit results (+210 and +473 cm^−1^). The 25% overestimate for the open form likely arises from the omission of dielectric screening by CHCl_3_ solvent in the vacuum TD-DFT calculations. A possible additional contribution from a small negative *i.e.* “*J*-type” *J*_CT_ term (≈−50 cm^−1^) was considered. This arises from a superexchange mechanism *via* a virtual CT state, and is defined as:^24^2
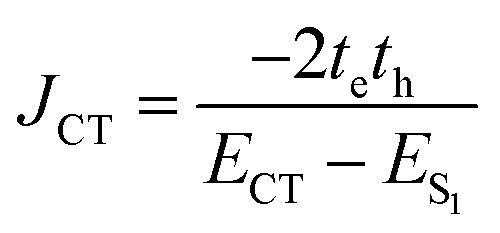
where *t*_e_ and *t*_h_ are electron and hole transfer integrals, sensitive to sub-Å chromophore displacements whilst *E*_CT_ − *E*_S_1__ is the energy gap between the CT and S_1_ states, assumed as +1500 cm^−1^ in agreement with literature on structurally-related PDI dimers.^24,39^ However, given the ≈0.7 nm PDI–PDI separation in the “open” geometry, which precludes significant overlap between frontier molecular orbitals (MOs), *J*_CT_ is expected to be minimal and was assumed to be negligible in this valPDI_2_ conformer.

In contrast, the closed conformer (0.35 nm PDI–PDI distance) populated in DMSO/water may support non-negligible short-range coupling (*J*_CT_). *t*_h_ and *t*_e_ for the “closed” conformer are +182 and −112 cm^−1^, respectively, and were obtained using CATNIP.^44^ Details of the method are given in Bressan *et al.*^24^ and in the SI. Using eqn (2), these values yield a *J*_CT_ value of +29 cm^−1^, an order of magnitude smaller than that determined for similar PDI structures with comparable interchromophore separation.^45,46^ The weak positive *J*_CT_ contribution to *J*_Tot_ in the closed conformer of valPDI_2_ is attributed to the ≈0.38 nm longitudinal slip between PDIs disrupting frontier MO overlap, consistent with calculations showing that such longitudinal displacement makes the product *t*_e_*t*_h_ negligible.^13,38^ A cofacial PDI dimer displaying large positive *J*_Coul_ and small negative *J*_CT_ has been reported by Hong *et al.*^47^ Therefore, even in the closed form, short-range coupling plays a marginal role in valPDI_2_, and changes in *J*_Tot_ are dominated by modulation of *J*_Coul_ and solvent dielectric screening effects.

Overall, the combined evidence from NMR, XRD, DFT, XTB and excitonic coupling analysis supports solvent-controlled switching between weakly coupled (“open”, CHCl_3_) and strongly coupled (“closed”, DMSO/water) valPDI_2_ conformers. We next investigate how these structural differences affect excited-state dynamics probed first by femtosecond transient absorption (fsTA) and then by half-broadband 2D electronic spectroscopy (HB2DES).

### Population dynamics

**Fig. 2:**
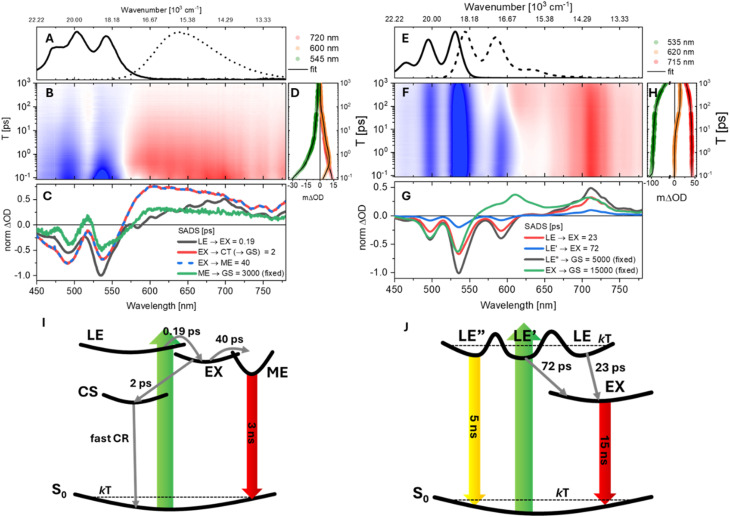
Solvent-dependent ultrafast photoinduced dynamics of valPDI_2_. (A) Normalised steady-state absorption (solid) and emission (dashed) spectra of valPDI_2_ in DMSO/water. (B) Broadband fsTA over 0.08–1000 ps pump-probe delay range for valPDI_2_ in DMSO/water. The intensity is given by 21 contours, negative signals (ground state bleach, GSB and stimulated emission, SE) are shown in blue and positive signals (excited state absorption, ESA) are shown in red. (C) Species-associated difference spectra (SADS) obtained by a target analysis of the data in (B), using the model shown in (I). (D) fsTA log time traces and fit of valPDI_2_ in DMSO/water at specific probe wavelengths. (E–H) Are the same of (A–D) for valPDI_2_ in CHCl_3_, with the target analysis using the model of (J).

The solvent-dependent population dynamics of valPDI_2_ were investigated by fsTA spectroscopy. The data are presented on a log *T* scale contour from 0.08 to 1000 ps (Fig. 2B and F). Data for refPDI and transient spectra at selected *T* values for valPDI_2_ in both solvents are provided in the SI (Fig. S4 and S5). Steady-state absorption and emission spectra of valPDI_2_ in DMSO/water and CHCl_3_ are reproduced in Fig. 2A and E for reference.

Following excitation in DMSO/water (Fig. 2B), valPDI_2_ shows an initial spectrum featuring a negative band between 450 and 590 nm due to ground-state bleach (GSB) and stimulated emission (SE), and a broad excited-state absorption (ESA) extending to the red edge of the probed spectral window. Within 1 ps, both the 535 nm GSB/SE and 700 nm ESA decay by ≈50% whilst a prominent ESA rises at 605 nm (Fig. 2D). Over tens of ps the ESA signal decays nonexponentially, as the GSB refills and a weak positive feature centred at 525 nm appears, which persists for longer than our time range (>1 ns, SI, Fig. S5).

In CHCl_3_ (Fig. 2F), valPDI_2_ exhibits a promptly formed broad negative feature attributed to overlapping structured GSB and SE. This band matches the inverted steady-state spectra. A positive ESA appears promptly at 700 nm (extending from 750 to 650 nm) and is nearly identical to that of refPDI (Fig. S4) and is thus assigned to an S_*n*_ ← S_1_ transition from the LE state. The spectra match similar data in the literature for related PDI molecules.^20,48,49^ The early time evolution of the open conformer of valPDI_2_ closely resembles that of refPDI under comparable conditions. However, over tens of ps, the SE (550–600 nm) partially recovers as a new ESA emerges between 600 and 650 nm (Fig. 2H). In contrast, refPDI shows nonexponential decay without any new ESA formation (SI, Fig. S4). After 100 ps valPDI_2_ exhibits ≈20% overall decay in signal amplitude, consistent with its ns fluorescence lifetime.

Such spectral evolution suggests branching within the excited state PES, not accounted for by a sequential model. Hence, we performed target analysis using GloTarAn^50^ (results of a global fit to a sequential model are reported in the SI). Four components were needed to yield an accurate fit to either dataset.

In DMSO/water, the components are *τ*_1–4_ = 0.19, 2, 40, and 3000 ps, whilst in CHCl_3_, *τ*_1–4_ = 23, 72, 5000 and 15 000 ps were required. Traces of the fits (solid black) are superimposed to the experimental traces in Fig. 2D and H. The ns lifetimes were constrained to values obtained by time-resolved fluorescence emission (Fig. 1C and D). Species Associated Difference Spectra (SADS) are shown in Fig. 2C (DMSO/water) and G (CHCl_3_).

In DMSO/water, the initial SADS (Fig. 2C, dark grey) exhibits a 0–0/0–1 intensity ratio that does not match the steady-state absorption. The negative band at 535 nm is stronger than the 0–1 GSB at 500 nm because it contains both GSB and monomer-like SE originating from the promptly populated LE state. Within 0.19 ps, this component forms a SADS characterised by ESA growth at 605 nm, decay at 700 nm, and quenching of SE at 535 nm. As PDI excimers display broad, nearly featureless ESA extending from ∼600 nm into the NIR,^4,20,21,51–54^ we assign this ultrafast evolution to LE → EX formation. The sub-ps timescale reflects the closed conformer geometry, which is preorganised for essentially barrierless population flow from LE to EX. Similar 100–200 fs EX formation has been reported for multiple cofacial PDI dimers by the Kim and Wasielewski groups, and by us for a bay-connected PDI dimer.^4,24,55,56^ EX formation in π-stacked PDI systems is governed by subtle geometric factors rather than by excitonic coupling strength alone. For example, in PDI nanowires^57^ and crystals^58^ a significant rotational displacement between chromophores can, in line with theory,^59^ suppress population of the EX minimum. In contrast, the closed conformer of valPDI_2_ combines a short π–π separation with negligible rotational (and transverse) displacements, features that enhance frontier MO overlap and promote structural relaxation along the π-stacking coordinate on the excited-state potential energy surface (*vide infra*). These structural characteristics promote ultrafast (vibrationally coherent) excimer formation in the “closed” conformer of valPDI_2_.

The EX state (red SADS) loses a substantial fraction of its initial ESA amplitude, accompanied by significant GSB recovery, with a 2 ps timescale. We attribute this rapid relaxation to symmetry-breaking charge separation (SBCS) *i.e.* formation of a PDI˙^+/^˙^−^ pair. SBCS is typically driven by dipolar solvation and should therefore occur on solvent-relaxation timescales. While bulk water solvation is complete within ∼2 ps, solvation in a 1 : 1 DMSO/water mixture is slower, extending over several ps, due to extended H-bond networks, as reported by Roy *et al.*^60^ We propose that the observed GSB refilling arises from SBCS followed by faster charge recombination (CS → GS). Because CR is faster than SBCS, the characteristic 615/710 nm PDI˙^+/^˙^−^ transients are not detected.^49,61^

The thermodynamic feasibility of photoinduced charge separation was evaluated using the Rehm–Weller formalism, which estimates the free energy change associated with electron transfer following photoexcitation Δ*G*_CS_, defined as:3
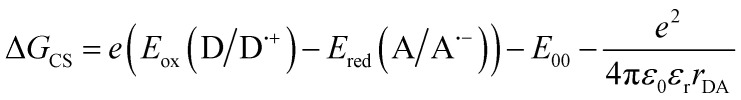
where *e* is the elementary charge, *E*_ox_(D/D˙^+^) and *E*_red_(A/A˙^−^) are the PDI oxidation and reduction potentials, respectively, and *E*_00_ is the excitation energy to the lowest singlet S_1_. The last term on the right-hand side of (3) accounts for the electrostatic interaction between the radical pair within valPDI_2_ and depends on the vacuum permittivity, (*ε*_0_) the relative dielectric constant of the 1 : 1 DMSO/water solvent (*ε*_r_), the centre-to-centre separation between the donor and acceptor moieties (*r*_DA_) *i.e.* the PDI–PDI distance.

For PDI, the one-electron half-wave oxidation and reduction potentials *versus* SCE have been reported by Lee *et al.*^62^ as +1.56 V and −0.55 V, respectively. Using these values, together with the experimentally determined *E*_00_ (2.36 eV), *ε*_r_ = 66, and *r*_DA_ = 0.35 nm (from XRD and XTB calculations, see above) yield a Δ*G*_CS_ = −0.282 eV, indicating thermodynamically favourable SBCS. The free energy of charge recombination can then be expressed as:

4 Δ*G*_CR_ = −(Δ*G*_CS_ + *E*_00_)

which gives Δ*G*_CR_ ≈ −2.05 eV. The strongly exergonic nature of this process provides a rationale for the fast CR following SBCS, preventing direct observation of the spectral features of the PDI˙^+/^˙^−^ pair.

A parallel, slower (40 ps) EX decay channel forms a SADS (green) whose distinctive lineshape in the 450–550 nm region is nm due to overlap of negative singlet GSB and positive T_*n*_ ← T_1_ absorption at ≈525 nm.^15,63–67^ This component develops far more rapidly than typical ns PDI intersystem crossing and is therefore assigned to singlet fission (SF). In this process, a non-negligible fraction of the EX populates a multiexciton ^1^(T_1_T_1_) state. As PDI has *E*(S_1_) = 2.36 eV and *E*(ME) ≈ 2 × *E*(T_1_) = 2.4 eV, ME formation is slightly endothermic, implying that thermal activation contributes to its relatively slow onset.^68^ Contributions from CT states close in energy to S_1_ can lower the ^1^(T_1_T_1_) energy through superexchange, enabling SF even when the ME state would be otherwise inaccessible.^4^ We propose that the highly polar DMSO/water environment (*ε*_r_ ≈ 66) increases the CT character of the EX state, as suggested by the fast CS channel, enhancing S_1_–ME mixing, thereby promoting SF in the closed conformer of valPDI_2_. EX-mediated ME formation over comparable timescales was reported for a slip-stacked terrylene diimide dimer by Margulies *et al.*,^69^ and a CT-enabled SF mechanism in pentacene dimers in polar benzonitrile was recently proposed by Thiel *et al.*^5^

The ME of valPDI_2_ persists for a few ns and, through vibronic intensity borrowing, produces the weak, red-shifted, and featureless fluorescence described earlier. Such long-lived emission indicates that the ME state does not dissociate, preventing quantification of the free triplet yield. In any case, such yield is expected to be modest, as a large fraction of the EX relaxes back to S_0_*via* CS followed by CR within less than 2 ps from photoexcitation. Notably, the FQY of the ME emission shows only a minor change beyond 50% water content (SI, Fig. S11) indicating that its formation and relaxation are primarily governed by solvent-induced conformational changes, with further increases in the dielectric constant of the environment having little influence on its behaviour.

We propose that the bifurcation within the EX PES originates from structural heterogeneity associated with the malonate ester linkers. The oxygen lone pairs can participate in intermolecular H-bonding with water, affecting both electrostatics and dimer geometry. Small variations in the PDI–PDI rotational displacement strongly affecting CT coupling, and in the long-axis slip coordinate, critical for SF,^37,39,70,71^ lead to a distribution of EX conformations for which the former or latter relaxation pathways are preferred. The photoinduced dynamics of valPDI_2_ in DMSO/water are summarised in Fig. 2I.

In CHCl_3_, both the excited-state timescales and spectral evolution differ markedly from those observed in the polar, protic medium. Upon photoexcitation, multiple spectroscopically indistinguishable minima of the LE PES are populated. These correspond to conformers arising from the flexible architecture of valPDI_2_. Such conformers are in thermal equilibrium in S_0_ but separated by barriers larger than *kT* in the excited state and are labelled in Fig. 2G as LE (red), LE′ (light blue), and LE″ (dark grey). From these minima, the population decays either directly to the ground state or to a long-lived component (green), assigned to the EX state based on its relatively flat ESA spanning 560–700 nm coupled to SE quench. We note that the long-lived SADS presents a peak at 615 nm which is not present in the data (see SI) and presumably arises from the fit compensating positive ESA and negative structured SE.

Although the PDI˙^+^ D_1_ ← D_0_ absorption also lies near 610 nm, the formation of a new ESA in this region cannot be attributed to SBCS: this mechanism would require a corresponding risetime at ∼710 nm, where the D_1_ ← D_0_ absorption of PDI˙^−^ contributes, but no such feature is observed.^49,61^ Instead, the strong positive signal at 700 nm, present from the earliest delay times and also observed for refPDI (SI), is assigned to the S_*n*_ ← S_1_ transition from the LE region of photoexcited valPDI_2_. The absence of any redshift or spectral reshaping of this band (see comparison with the absorption spectrum of chemically reduced refPDI in Fig. S5) confirms that PDI˙^−^ is not formed. The data therefore support EX formation rather than SBCS. Further, EX formation aligns with the weak, long-lived 750 nm emission, which a dark CS state fails to account for.

The fastest component (red, Fig. 2G) corresponds to a subset of the LE population that converts to EX with a 23 ps timescale. Over ∼72 ps, a smaller LE′ subpopulation (blue) also relaxes to EX. A substantial fraction of the initially excited population (dark grey) resides in LE″ and does not reach the EX region; instead, it decays radiatively back to S_0_ over a few ns, consistent with the LE lifetime of refPDI in CHCl_3_. The EX state persists for ∼15 ns, in agreement with the long-lived weak fluorescence observed at 750 nm for valPDI_2_ in CHCl_3_ (Fig. 1C). This suggests that over several ps, the LE and LE’ macrocycle subpopulations adopt a “closed” conformation expelling the intercalated CHCl_3_ solvent molecules.

Because SADS1-3 are spectrally identical, their relative amplitudes directly reflect the relative populations of the corresponding LE subsets, *i.e.* conformers. From this, we conclude that a branching mechanism governed by conformational heterogeneity causes more than 60% of photoexcited valPDI_2_ (LE″, dark grey) to relax radiatively to S_0_, producing monomer-like structured fluorescence and SE, whereas the remaining fraction (red and blue SADS) undergoes LE/LE′ → EX conversion on tens of ps timescales. The full photoinduced dynamics for valPDI_2_ in CHCl_3_ are summarised in Fig. 2J.

Overall, the fsTA measurements show that the excited state dynamics of valPDI_2_ proceed through conformation-dependent branching pathways. In DMSO/water, the collapsed conformer enables rapid, sub-ps EX formation, a fraction of which forms a ME state, whist most of the population quickly refills the ground state *via* CS followed by (faster) CR. In CHCl_3_ the open conformers undergo slower structural dynamics, leading to a mixture of monomer-like and EX excited state populations. The sub-ps EX formation observed in the collapsed conformer suggests that vibrationally coherent dynamics may assist the system in escaping the LE region of the excited-state PES. We now investigate with HB2DES whether coherent nuclear-wavepacket motion influences the ultrafast photophysics of valPDI_2_.

### Coherent vibrational dynamics

We employed HB2DES to investigate the potential role of S_1_ vibrational coherence in the excited state dynamics of valPDI_2_. The waiting time *T* (analogous to the pump-probe delay time in fsTA) was scanned in 10 fs steps between 0 and 1200 fs to capture ground and excited state wavepacket motion, detected as amplitude oscillations during *T* superimposed to the population dynamics. From a Fourier transform (FT) of these data we construct a 3D spectrum, which is then “sliced” at frequencies of specific resonance Raman active molecular vibrations to create their “beatmaps”. Beatmaps are thus a full frequency domain representation of nuclear wavepacket dynamics in which the contribution due to a specific molecular vibration is resolved as a function of the excitation (pump, *ν̃*_1_) and detection (probe, *ν̃*_3_) dimensions and with respect to its beat frequency and phase over *T*. Details of the HB2DES beatmap construction are presented in the SI (Fig. S7).

**Fig. 3:**
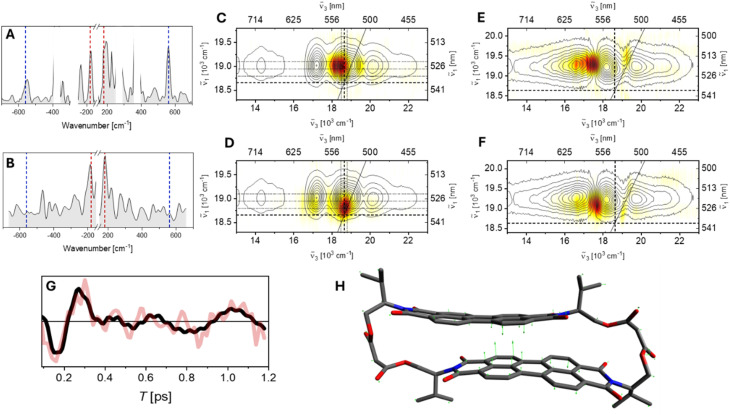
Solvent-dependent vibrational coherences in valPDI_2_. Rephasing summary Raman spectra of valPDI__2__ in CHCl_3_ (A) and in DMSO/water (B) shown as grey shaded areas. Strong solvent Raman modes are masked by white rectangles. Red and blue dashes indicate the out-of-plane bending mode at 180 cm^−1^ and the PDI core breathing mode at 550 cm^−1^, respectively. (C and D) Rephasing positive and negative beatmaps of the 180 cm^−1^ Raman active mode of valPDI_2_ in CHCl_3_, respectively. (E and F) Same as (C and D) for DMSO/water. Beatmaps are shown as white–yellow–red heatmaps and are all normalised to unity. Contour lines showing the real part of the corresponding absorptive HB2DES spectra (*T* = 200 fs) are overlaid. (G) Integrated residuals of a global fit to the real part of the rephasing HB2DES of valPDI_2_ in DMSO/water. The residuals were integrated over 300 × 300 cm^−1^ squares centred at *ν̃*_1_ = 19 200 cm^−1^; *ν̃*_3_ = 17 400 cm^−1^. Overlaid (black solid line) is a fit to a sum of damped cosines (90, 180 and 670 cm^−1^, latter is a DMSO frequency), yielding a ≈180 fs dephasing time for the 180 cm^−1^ frequency. (H) Displacement vectors of the 180 cm^−1^ out-of-plane bending mode contracting the PDI–PDI distance in the closed conformer of valPDI_2_. H atoms removed for clarity.

Our experimental implementation of HB2DES enables the retrieval of both the real and imaginary components of the rephasing (photon-echo) and non-rephasing (free induction decay) signals. Acquiring the full complex-valued response is necessary for the unambiguous assignment of vibrational coherence to either the electronic ground (S_0_) or excited (LE, S_1_) state PESs, which is based on the sign (*i.e.* the phase) of the oscillatory response following complex FT. The discussion below is restricted to the rephasing data, as the nonrephasing response does not here contain additional information. For rephasing signals, positive coherence frequencies correspond to density matrices |s_*n*_〉〈s_*n*+1_| evolving during *T*, (where s is an arbitrary electronic state and *n* ≥ 0 is the number of vibrational quanta of a resonance Raman active vibrational mode) and encode contributions from wavepackets evolving in S_0_ and S_1_, whereas negative frequencies, corresponding to |s_*n*+1_〉〈s_*n*_| density matrices exclusively arise from vibrational wavepacket evolution on the excited state potential energy surface. It should be emphasised that this correlation is rigorously valid only for vibrational mode energies significantly exceeding *kT*, as discussed below. The selection of specific vibrational wavenumbers for further analysis in beatmaps is guided by the inspection of the rephasing vibrational spectra integrated over the excitation (*ν̃*_1_) and detection (*ν̃*_3_) frequency axes (“summary Raman”) shown in Fig. 3A and B for valPDI_2_ in CHCl_3_ and DMSO/water respectively.^72,73^

The summary Raman spectrum of valPDI_2_ in CHCl_3_ (Fig. 3A) reveals a few resonantly enhanced vibrational modes between 100–600 cm^−1^, along with nonresonant CHCl_3_ solvent contributions at 261 and 369 cm^−1^ (masked in white). In PDI dimers, modes in the 100–400 cm^−1^ range correspond to delocalised PDI and scaffold deformations. DFT calculations and the absence of signals in this spectral region for refPDI support assignment of the strong ±180 cm^−1^ signals (red dashes) to an out-of-plane (OOP) PDI bending mode (Fig. 3H). The 300–600 cm^−1^ region corresponds to PDI ring breathing modes, with the ±550 cm^−1^ peaks (blue dashes) attributed to a strongly Raman allowed ring breathing vibration, confirmed *via* DFT, literature, and comparison with refPDI.^14^ Both 180 and 550 cm^−1^ modes contribute to the negative side of the Raman spectrum, indicating S_1_ activity.^73,74^ However, their presence on the positive side suggests ground state wavepackets also contribute. To confirm the frequencies observed in the summary Raman of valPDI_2_ in CHCl_3_, we fitted the time-domain oscillations of rephasing HB2DES residuals (integrated over a 300 cm^−1^ square centred on the GSB + SE feature maximum) to a sum of damped cosines (Fig. S8). The 550 cm^−1^ and 180 cm^−1^ components are damped with a ≈650 fs rate, a timescale typical of vibrational dephasing for chromophores in solution.^75^ The strong CHCl_3_ solvent mode at 359 cm^−1^, with dephasing time fixed to 1 ps, was included to yield a good fit.

The amplitude ratio of rephasing positive to negative peaks reflects the total number of allowed Liouville-space pathways containing oscillatory terms of the type |s_*n*_〉〈s_*n*+1_| *vs.* |s_*n*+1_〉〈s_*n*_|.^73,76,77^ For the 550 cm^−1^ mode, this ratio is ≈2, consistent with the displaced harmonic oscillator (DHO) model. Beatmaps relative to this mode can be fully assigned following the DHO model and are reported and discussed in the SI (Fig. S8). As a reference, the rephasing ±550 cm^−1^ beatmaps of refPDI are also reported in Fig. S10. In contrast, the 180 cm^−1^ mode shows a ≈1 : 1 ± amplitude ratio, diverging from DHO expectations. We address this point below.

Rephasing positive and negative beatmaps of the 180 cm^−1^ mode of valPDI_2_ in CHCl_3_ (Fig. 3C and D) show that the positive features are blue-shifted along *ν̃*_1_ by (at least) one quantum of vibrational excitation relative to their negative counterpart, located at the 0–0 transition. Comparison with HB2DES absorptive data (black contours, reproduced from Fig. S6) shows coherence amplitude contributions, along *ν̃*_3_, between 17 800 and 18 900 cm^−1^ consistent with GSB (+180 cm^−1^) and SE (±180 cm^−1^) pathways. Absence of amplitude at *ν̃*_3_ = 14 300 cm^−1^ (S_*n*_ ← S_1_ LE ESA) implies minimal displacement along this mode between S_1_ and higher excited states. Hence, the coherent vibrational response of the 180 cm^−1^ mode of valPDI_2_ in CHCl_3_ can be fully rationalised by a two-level DHO model including the S_0_ and LE PESs.

For such a model system, GSB and SE coherence amplitudes are both governed by the displacements between the S_0_ and S_1_ (LE) PESs. Hence, it is unphysical for a vibrational mode to be S_0_ silent while being active in S_1_. Nonetheless, the nearly 1 : 1 amplitude ratio observed in the summary Raman spectrum in Fig. 3A suggests S_1_ only activity. This apparent inconsistency can be explained by thermal population of vibrational levels within *kT* (≤200 cm^−1^) at room temperature. Assuming that thermal population is significant only for *v* = 1, four additional ‘anti-Stokes-like’ vibrationally coherent rephasing GSB pathways must be considered, originating from thermally populated |*g*_1_〉〈*g*_1_| and beating at negative frequency during *T*, as shown by Green *et al.*^77^ The corresponding double-sided Feynman diagrams (DSFDs) are provided in the SI (Fig. S9). We refer to such pathways as hot ground state bleach (HGSB) pathways. These shift spectral weight from positive to negative peaks of the integrated vibrational spectrum according to a Maxwell–Boltzmann distribution, flattening the amplitude ratio of the low frequency region of the summary Raman spectrum. HGSB contributions also broaden beatmap features due to increased numbers of possible quasi-degenerate DSFDs involving multiple thermally populated states excited by the pump, as we have previously reported.^24,78^

Thus, the vibrational response of valPDI_2_ in CHCl_3_ is fully explained by a two-state DHO model incorporating HGSB pathways for the 180 cm^−1^ mode. These results do not therefore suggest involvement of coherently excited nuclear motion in S_1_ in the formation of the valPDI_2_ excimer. This is expected, as the main LE → EX timescale (23 ps, Fig. 2G) is ≈40 times slower than the 550 cm^−1^ vibrational dephasing time (650 fs) retrieved from the damped cosine fit.

In DMSO/water, valPDI_2_ exhibits different vibrational features to the dimer or monomer (refPDI, Fig. S10) in CHCl_3_. Its summary Raman spectrum (Fig. 3B) retains the ±180 cm^−1^ OOP bending mode observed in CHCl_3_ but lacks the ±550 cm^−1^ ring breathing mode. These differences likely arise from the “open” and “closed” conformers having different displacements along these normal mode coordinates and differing electronic transitions, consistent with the observed differences between their electronic spectra, affecting resonance enhancement.^79,80^ Coherent vibrational responses dominated by low frequency vibrations were previously reported, among others, by Kim *et al.* and O'Connor *et al.* for EX forming cofacial PDI dimers^16,19^ and by us in a more rigid macrocyclic PDI dimer.^24^

The ±180 cm^−1^ beatmaps in DMSO/water (Fig. 3E and F) resemble those in CHCl_3_ along *ν̃*_1_, but differ along *ν̃*_3_: amplitude is localised near the 0-crossing between GSB and ESA and extends toward the EX ESA at 16 300 cm^−1^. Beatmap amplitude localised in such region suggests contributions from ESA pathways *i.e.* vibrational coherence in the S_1_ PES. The lack of GSB features suggests minimal S_0_–S_1_ displacement along this coordinate for the strongly coupled valPDI_2_ conformer. This implies the S_0_–S_1_ displacement is insufficient to generate appreciable GSB coherence amplitude (which scales with the 4^th^ power of the S_0_–S_1_ displacement along this vibrational coordinate) but large enough to launch S_1_ wavepacket motion modulating the ESA band, dependent on the product of squared displacements between S_0_–S_1_ and S_1_–S_*n*_.^73,78^ This suggests that the 180 cm^−1^ mode is significantly displaced along S_1_–S_*n*_ for the “closed” conformer but only weakly along S_0_–S_1_, in contrast to the “open” conformer.

The OOP PDI core bending coordinate corresponds to a contraction of the interchromophore spacing and hence promotes ultrafast, vibrationally coherent, EX formation, revealed by the dephasing time of such vibration matching the EX formation timescale.^16,24,81^ Therefore, we analysed time-domain oscillations of rephasing HB2DES residuals (integrated over a 300 cm^−1^ side square centred at 19 250, 16 950 cm^−1^) using damped cosine fits, as shown in Fig. 3G. The 180 cm^−1^ component is damped with a 180 fs dephasing time, a timescale closely matching the 190 fs EX formation. The ps-lived modes at 90 and 670 (DMSO solvent) cm^−1^ were needed to yield a good fit. The similar vibrational dephasing and EX formation timescales suggest a role for the 180 cm^−1^ OOP bending mode in enabling efficient LE-to-EX population transfer promoted *via* reduction of the PDI–PDI interchromophoric coordinate, as previously shown for structurally related PDI dimers.^16,19,24,25,56^

## Conclusions

Ultrafast coherent spectroscopies reveal that the photophysics of a macrocyclic perylene diimide dimer, valPDI_2_, are dictated by a pronounced solvent-induced conformational switch that modulates both excitonic coupling and excited-state relaxation. In weakly polar CHCl_3_, valPDI_2_ adopts an “open” macrocycle geometry with a large PDI–PDI separation, yielding weak H-type coupling and steady-state spectra closely resembling those of monomeric refPDI. Under these conditions, photoexcitation largely populates a monomer-like local excited state, while conformational heterogeneity enables a fraction of the local excited state population to form an excimer over several tens of ps. Half-broadband two-dimensional electronic spectroscopy further shows that vibrational coherences in the “open” conformer follow a simple displaced-harmonic-oscillator description of the ground and excited state potential energy surfaces, with no evidence for coherent nuclear motion funnelling population toward the excimer region of the excited state, in agreement with its slow formation.

In sharp contrast, in polar DMSO/water the dimer collapses into a tightly π-stacked cofacial conformer characterized by strong H-type coupling and broadened, red-shifted emission. This “closed” geometry is preorganised to support sub-200 fs barrierless excimer formation, after which the excimer partitions between rapid charge separation and recombination and slower multiexciton generation *via* excimer-mediated singlet fission. The weakly emissive multiexciton state persists on the nanosecond timescale without dissociating into free triplets. Half-broadband two-dimensional electronic spectroscopy reveals that, unlike in CHCl_3_, the dominant coherence corresponds to an out-of-plane 180 cm^−1^ PDI bending mode with a dephasing time (∼180 fs) in close agreement with the excimer formation timescale. Localisation of the coherence amplitude within the ESA region further indicates wavepacket motion in the excited state potential along a nuclear coordinate that contracts the PDI–PDI separation, thereby supporting vibrationally coherent LE → EX population transfer.

Importantly, our results demonstrate that in the strongly coupled structure, dimer conformation promotes excimer formation and solvent polarity enhances its charge transfer character, opening a channel for multiexcition state generation. However, the stabilisation of the radical pair state also accelerates relaxation to the ground state *via* ultrafast charge separation and recombination, creating a competing decay pathway. Optimising multiexciton generation, and therefore triplet yield, requires striking a balance: polarity-driven conformational tuning must be sufficient to promote short-range coupling and thus charge transfer-assisted excimer-to-multiexciton conversion, but not so strong as to favour fast charge separation/recombination pathways that short-circuit multiexciton formation yield.

## Experimental

### Synthesis

The macrocycle dimer valPDI_2_ and the monomer refPDI were prepared and characterised as previously described.^27^

### fsTA and HB2DES

Ultrafast spectroscopy data were acquired using a previously reported home-built actively referenced fsTA/HB2DES spectrometer^72,82^ in 1 mm optical path static fused silica cells (Starna), with the concentration of valPDI_2_ adjusted to a peak OD of 0.5 in CHCl_3_ and 0.3 in DMSO/water. Sample and solvents were used as received. Briefly, 500 µJ pulses from the output of a Ti:Sa regenerative amplifier (Spitfire Ace, Spectra-Physics) operating at 800 nm and 1 kHz repetition rate pump a commercial noncollinear optical parametric amplifier (NOPA, Topas White, Light Conversion) to generate pulses centred at 520 nm (19 200 cm^−1^). The broadband visible pulse is pre-compressed by a commercial folded grism compressor (Fastlite) to achieve close to FT limited pulses at the sample position. Downstream, a commercial acousto-optical programmable dispersive filter (AOPDF, Dazzler, Fastlite) creates a pair of pump pulses with programmable time delay (coherence time *τ*) and relative carrier-envelope phase. *τ* is scanned shot-to-shot from −95 to 0 fs in 0.792 fs steps. Real and imaginary parts of the rephasing and nonrephasing responses are obtained by a 3 × 1 phase-cycling method^83^ and summed to obtain absorptive 2D spectra. Each 2D spectrum is averaged over 270 laser shots per *τ* value. The waiting time delay *T* between the second pump and the probe is introduced with a retroreflector mounted on a mechanical linear translation stage (Physik Instrumente). Data are measured with 10 fs steps from 0 to 1200 fs to record vibrationally coherent dynamics (HB2DES) or at increasing *T* steps between −1500 fs and 1 ns to recover population dynamics (fsTA). Pump scatter and fluorescence contributions are removed by subtracting a 2DES spectrum acquired at *T* < 0 fs. The probe pulse (WLC) is generated by focusing ≈1 µJ of the 800 nm regenerative amplifier output into a 3 mm static sapphire plate and spans 13 500–23 000 cm^−1^. Two pairs of dispersive mirrors at 5° and 19° angles of incidence (PC 1332, Ultrafast Innovations) recompress the WLC which is split before the sample by a 50 : 50 beamsplitter to provide probe and reference. The probe is crossed at 4° with the collinear pump pair at sample position. Pump and probe spot sizes are 80 and 160 µm, respectively. Pump pulse energy and duration at sample position are ≈300 nJ and 30 fs, respectively. The signal is recollimated after the sample and the signal and reference are focused into a home-built dual channel prism-based spectrometer and recorded shot-to-shot by a pair of 1024 pixels CCD detectors (Stresing) synchronised to the AOPDF. The signal is referenced using an active noise reduction method proposed by Feng *et al.*^84^ The instrument response function (*ca.* 45 fs) is measured by spectrally resolving the instantaneous nonresonant solvent response. All measurements were conducted at the magic angle to rule out contributions from rotational diffusive motion to the measured signal decay. HB2DES beatmaps are obtained by a procedure previously discussed in the literature and outlined in the SI (Fig. S6).^72,73,76^

### Computational methods

Crystallographic structures^27^ of the “open” and “closed” valPDI_2_ were solvated with CHCl_3_ (25 explicit) and H_2_O (40 explicit) using the SOLVATOR algorithm as part of the XTB package in ORCA 6.1.0.^30,35^ After solvent optimisation using molecular dynamics trajectories, the solvated structures were reoptimised using XTB. A series of 10 molecular dynamics trajectories using random seed velocities from a Wigner distribution for the open structure were performed at 300 K using canonical sampling velocity rescaling (CSVR) out to 200 ps in 0.5 fs steps to assess the average solvent occupancy of the cavity.^85^ It is not feasible to perform long-timescale simulations with only 40 water molecules due to the strong intermolecular hydrogen-bonding interactions inherent to water. Accurately capturing bulk-like behaviour in water would require hundreds of explicit molecules. In contrast, for chloroform, thermal energy at ambient temperatures is sufficient to overcome intermolecular forces. As a result, simulations with 25 chloroform molecules were found to yield reliable trajectories.

## Author contributions

Giovanni Bressan: conceptualisation; methodology; investigation; formal analysis; writing – original draft; writing – review and editing; funding acquisition; supervision. Denis Hartmann: investigation; methodology; resources; writing – review and editing. Jonathan Brouwer: investigation; methodology; writing – review and editing. Erico M. Braun: formal analysis; methodology; writing – review and editing. James N. Bull: software; formal analysis; writing – review and editing. Timothy A. Barendt: conceptualisation; funding acquisition; methodology; resources; supervision; writing – review and editing.

## Conflicts of interest

There are no conflicts to declare.

## Note added after first publication

This version replaces the manuscript published on 22^nd^ January 2026 which contained an incorrect version of eqn (3). The RSC apologises for any confusion.

## Supplementary Material

SC-017-D5SC09512C-s001

## Data Availability

All the data that support the findings of this study are available in this article and its supplementary information (SI). The raw data are available from the corresponding authors upon request. Supplementary information: structure of reference PDI, refPDI ns fluorescence, discussion and calculations of solvent-dependent excitonic coupling, refPDI fsTA in CHCl_3_ and DMSO/water, fsTA and global fit analysis of valPDI_2_ in CHCl_3_ and DMSO/water and chemically reduced radical anion absorption, HB2DES of valPDI_2_ in CHCl_3_ and DMSO/water, scheme of the beatmap calculation method, valPDI_2_ 550 cm^−1^ ring breathing coherence analysis, double-sided Feynman diagrams for hot ground state bleach, refPDI rephasing beatmaps of the 550 cm^−1^ mode, fluorescence quantum yields *vs.* water/DSMO volume ratio, coordinates. See DOI: https://doi.org/10.1039/d5sc09512c.
