# Supplementary material for: Conformational switching modulates excited-state pathways in a cofacial perylene dimer
Source: Chem Sci. 2026 Jan 22;17(12):5921–33. doi: 10.1039/d5sc09512c (PMC12851298; doi:10.1039/d5sc09512c)
Supplement: SC-017-D5SC09512C-s001 [file SC-017-D5SC09512C-s001.pdf]

Supplementary Information (SI) for Chemical Science.
This journal is © The Royal Society of Chemistry 2026

SUPPLEMENTARY INFORMATION

Conformational Switching Modulates Excited-State Pathways in a Cofacial Perylene Dimer

[1,*]Giovanni Bressan, [2]Denis Hartmann, [1]Jonathan Brouwer, [1,3]Erico M. Braun, [1]James N. Bull and [2,*]Timothy A. Barendt

[1]School of Chemistry, University of East Anglia, Norwich NR4 7TJ, United Kingdom

[2]School of Chemistry, University of Birmingham, Birmingham B15 2TT, United Kingdom

[3]Instituto de Fisica, Universidade Federal do Rio Grande do Sul, Porto Alegre, Av. Bento Gonçalves, 9500, Brazil

Authors for correspondence: g.bressan@uea.ac.uk; t.a.barendt@bham.ac.uk

Structure of reference PDI monomer 3

Nanosecond fluorescence of the refPDI monomer 4

Discussion of solvent-dependent excitonic coupling and calculations 5-6

fsTA and global analysis of refPDI monomer in $CHCl_3$ and DMSO/water 7

fsTA and global fit analysis of valPDI$_2$ in $CHCl_3$ and DMSO/water + radical anion 8

Absorptive HB2DES spectra of valPDI$_2$ in $CHCl_3$ and DMSO/water 9

Scheme of the beatmap calculation method 10

valPDI2 550 cm^{-1} ring breathing coherence analysis 11

Double-sided Feynman diagrams for hot ground state bleach rephasing negative 12

refPDI rephasing beatmaps of the 550 cm^{-1} mode 13

Fluorescence quantum yields vs water/DSMO volume raio 14

Coordinates 15

References 34

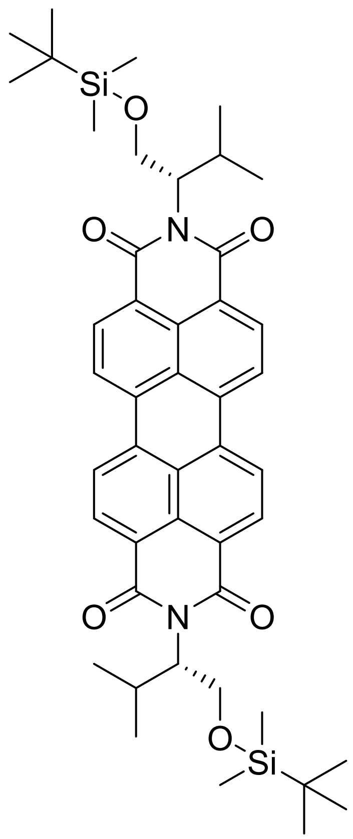

Figure S 1 Molecular structure of the **refPDI** monomer

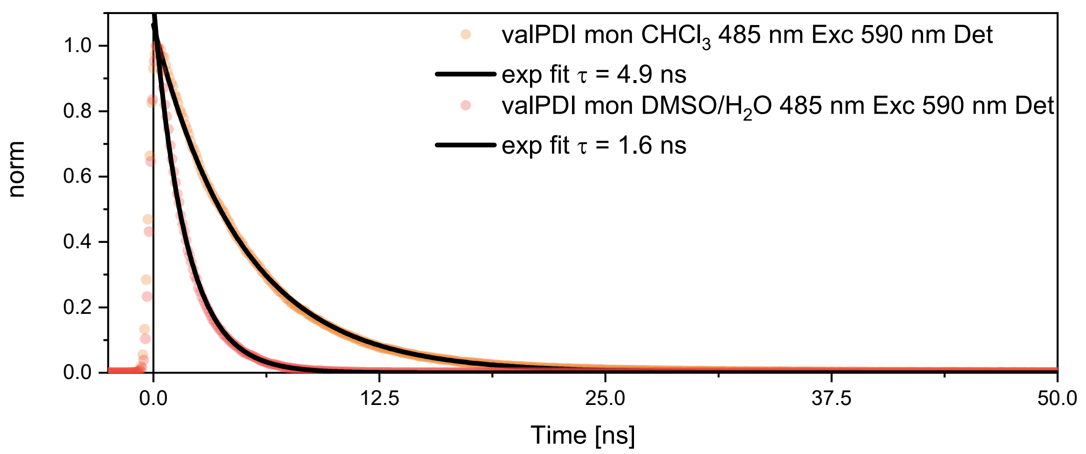

Figure S 2 ns fluorescence traces and fit (black) of **refPDI** in $CHCl_3$ (orange) and 1:1 DMSO/water (red)

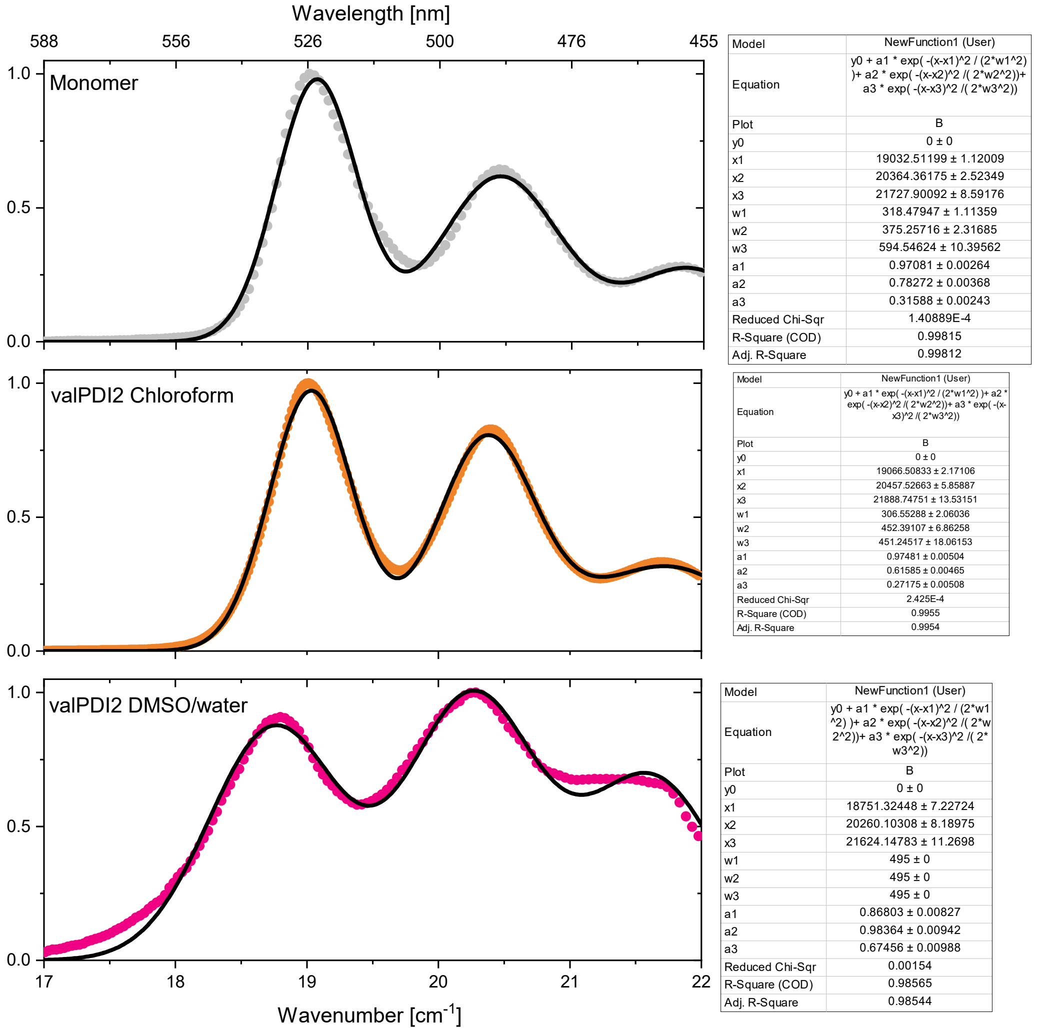

Figure S 3 steady-state fits for Spano's excitonic coupling model

Details of the excitonic coupling calculations

The excitonic coupling strength for **valPDI₂** in chloroform and DMSO/water can be determined from their steady-state absorption spectra, if the Huang-Rhys factor $\lambda^2{}_{mon}$ of the reference monomer refPDI is known.[1–3] $\lambda^2{}_{mon}$ is defined as:

$$\lambda^2{}_{mon} = \left(R_{0-1}/R_{0-0}\right) \qquad \text{(S1)}$$

Where R_{0-0} and R_{0-1} are the areas of the 0-0 and 0-1 vibronic bands of its steady-state absorption spectrum (see Figure S3). $\left(R_{0-1}/R_{0-0}\right) = 0.68$ for refPDI. Equation S1 can then substituted into Equation S2 which gives the total excitonic coupling when solved for J_{Tot}:

$$R_{abs} = \left(\lambda^2{}_{mon}\right)^{-1}\left[\frac{1 - G(0,\lambda^2)e^{-\lambda^2{}_{mon}J_{Tot}/\omega_0}}{1 - G(1,\lambda^2)e^{-\lambda^2{}_{mon}J_{Tot}/\omega_0}}\right]^2 \qquad \text{(S2)}$$

Where $R_{abs} = \left(\lambda^2{}_{dim}\right)^{-1} = (R_{0-0}/R_{0-1})$ of **valPDI₂** in chloroform or DMSO/water and $\omega_0$is the spacing of the vibronic progression (1350 cm^{-1}). Finally, G is the vibrational function, as defined in Equation S3:

$$G\left(v_t;\lambda^2\right) = \sum_{\substack{u=0,1,..\\ u \neq v_t}} \frac{\left(\lambda^2\right)^u}{u!\left(u - v_t\right)}, v_t \in N_0 \tag{S3}$$

The values of J_{Tot} extracted using the method outlined above are reported in the main manuscript.

The long-range Coulombic coupling for both conformers was calculated using the transition electrostatic potential method (trESP) as implemented in Multiwfn. This approach relies on point atomic charges rather than TDMs and yields more reliable estimation of coupling strengths when dipole-dipole distances are comparable or smaller to the molecular dimensions. The sum over the electrostatic interactions between transition charges of the PDI pair yields the long-range coupling J_{Coul}, as per Equation S4:

$$J_{Coul} = \frac{1}{4\pi\varepsilon_0}\sum_i\sum_j \frac{q_i^{(1)}q_j^{(2)}}{\left|r_i^{(1)} - r_j^{(2)}\right|} \tag{S4}$$

In which ε_0 is the permittivity of free space, $q_i^{(1)}$ is the transition charge of the *i*-th atom and $r_i^{(1)}$ is the position vector associated to the *i*-th charge. We used time dependent (TD-)DFT at the ω-B97XD level of theory and dgdzvp as basis set in Gaussian 16 to calculate the transition densities of the two conformers of **valPDI₂** populated in chloroform and DMSO/water. The transition densities were fit to atomic partial charges and the Coulomb coupling terms were calculated at the optimised ground state structures. The atomic partial charges were all rescaled by $\sqrt{2}$ as indicated in the Multiwfn manual.

The short-range couplings were calculated by using the charge transfer integral package CATNIP, the molecular orbitals (MO) coefficients were obtained by DFT calculations at the ω-B97XD/dgdzvp level of theory in Gaussian 16.

Both the long- and short-range coupling calculations required to determine transition charges or MO coefficients of each PDI chromophore. Such fragments were obtained by disconnecting the dimer structures at two opposite alpha carbons between the pair of C=O of each ligand to yield two identical "halves", followed by capping with H-atoms at the broken bonds positions to keep the S_0 multiplicity. The added H atoms were not included in the fragments used for the long-range coupling calculation in Multiwfn. Both dimer structures were geometry-optimized followed by frequency calculations to prove the existence of true minima, as determined by the absence of imaginary frequencies. All relevant coordinates are reported at the end of the present document.

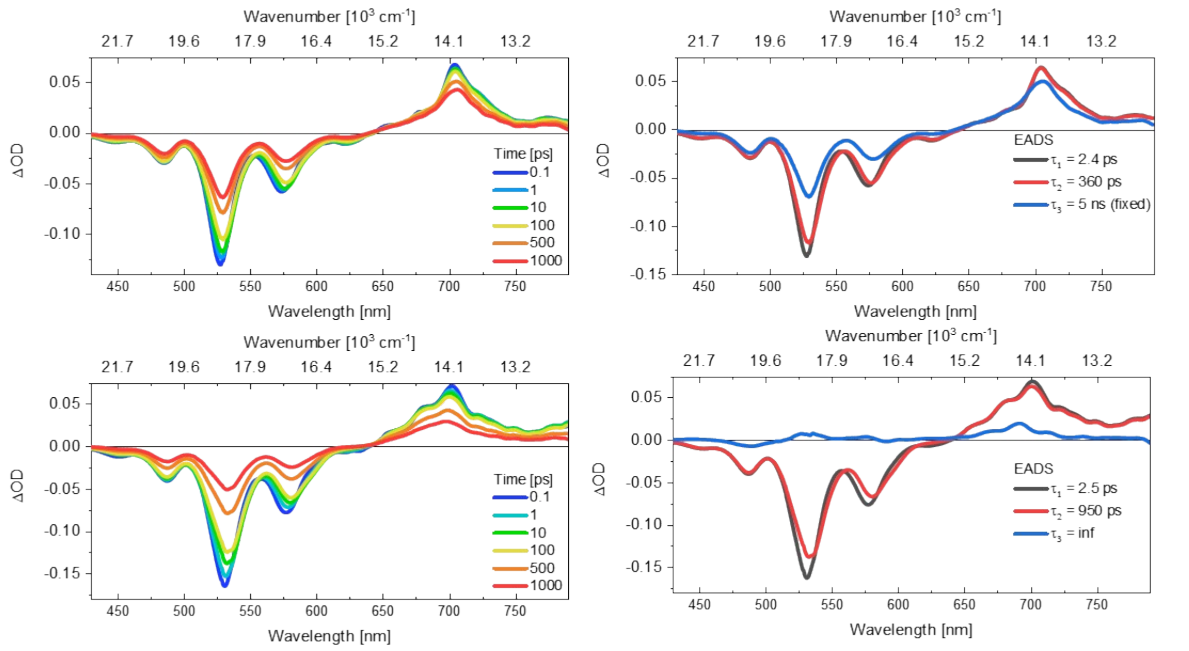

Figure S 4 fsTA spectra at selected pump probe delay times (left) and EADS from global fitting (right) of **refPDI** monomer in $CHCl_3$ (top) and DMSO/water (bottom)

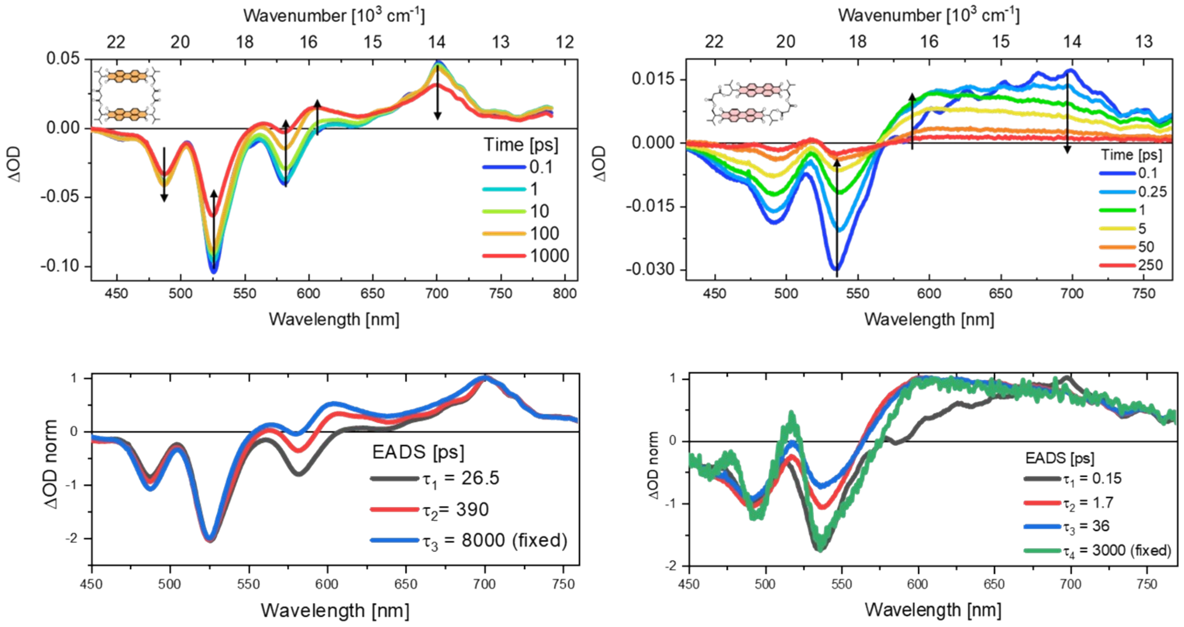

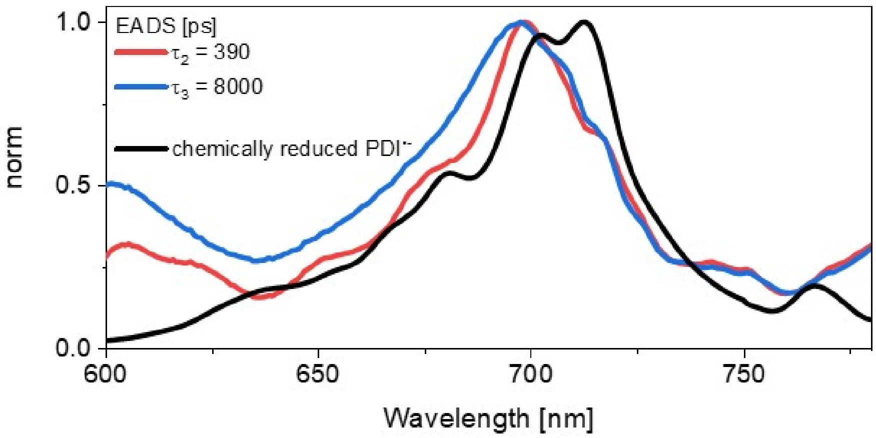

Figure S *5 (*top*)* fsTA spectra at selected pump probe delay times of **valPDI$_2$** in $CHCl_3$ (left) and DMSO/water (right).

(middle) normalised evolution associated decay spectra (EADS) obtained by applying a sequential model to the fsTA data of **valPDI$_2$** in $CHCl_3$ (left) and DMSO/water (right). Data were fit in GloTarAn.[4] Global fit required 3 and 4 components -indicated in the legend- to accurately fit the data in chloroform and DMSO/water, respectively.

(bottom) comparison between ESA ($S_n \leftarrow S_1$) region of normalised EADS2 and EADS3 of **valPDI$_2$** in $CHCl_3$ (maxima at 700 nm) and steady-state absorption of the chemically reduced **refPDI** radical anion (maximum at 710 nm). The radical anion was produced adding 0.3 ml of a saturated solution of sodium dithionite ($Na_2S_2O_4$) to 3 ml of a 0.2 mM THF solution of **valPDI$_2$**

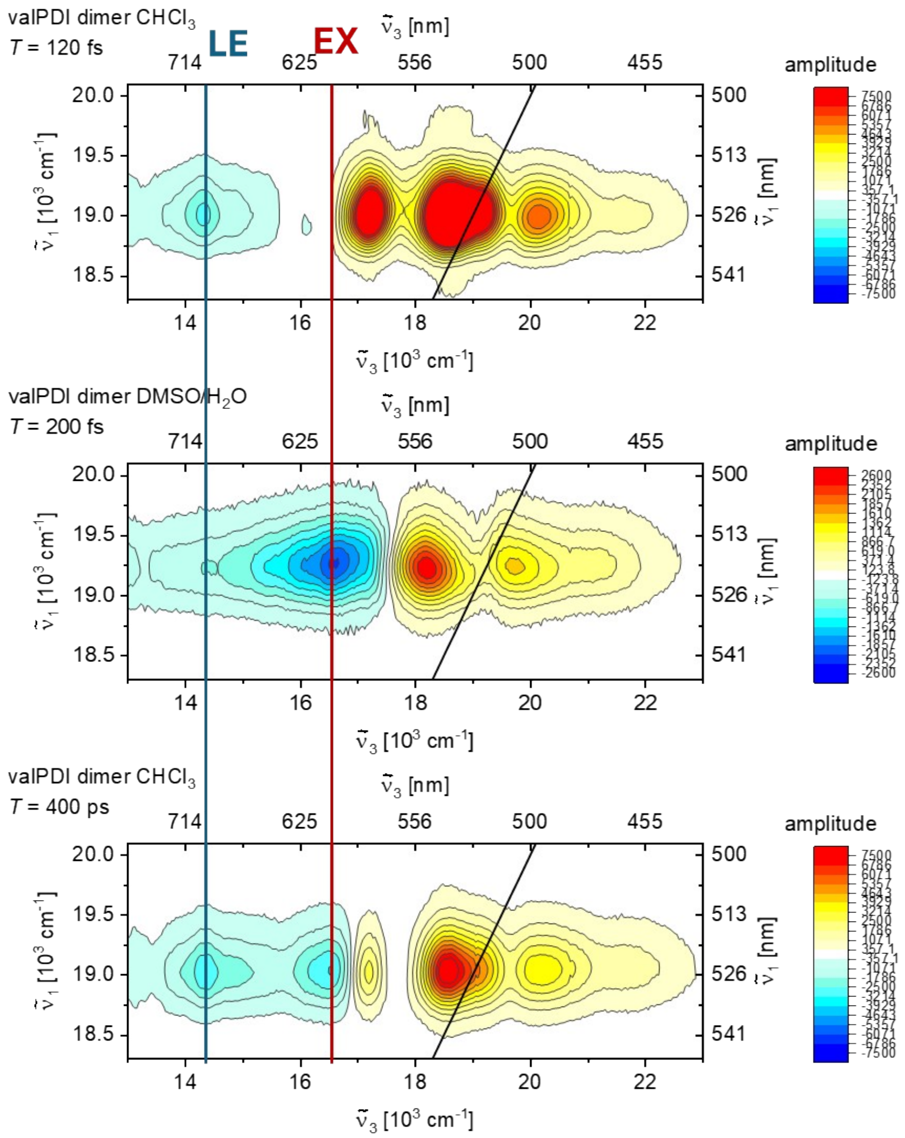

Figure S 6 Absorptive HB2DES of **valPDI$_2$** in $CHCl_3$ and DMSO/water showing the superposition of EX (red) and LE (blue) states in the open conformation.

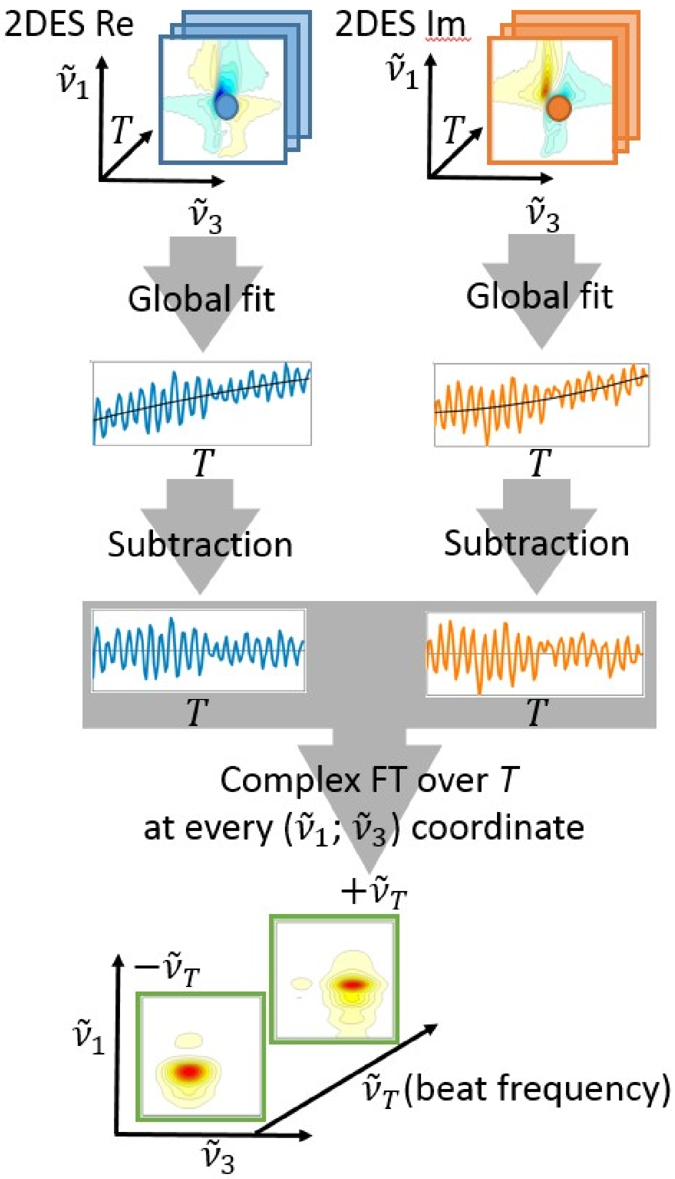

Figure S 7 Scheme of the method to extract complex valued coherent beatmaps from a HB2DES dataset. Adapted from Bressan et al.[5]

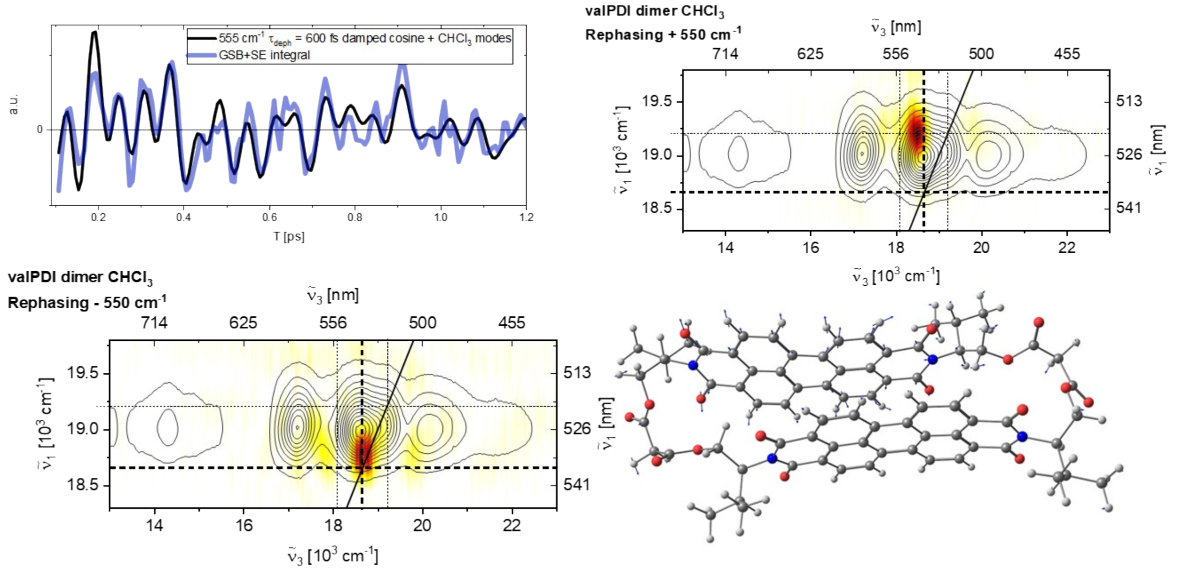

Figure S 8 Integrated oscillatory residuals (blue) in the GSB+SE region of the **valPDI$_2$** response in CHCl3. Data were fit to a sum of damped cosines at 369 cm^{-1} with fixed dephasing time of 1 ps to account for the strong $CHCl_3$ solvent response and to molecular modes at 180 and 550 cm^{-1} with 650 fs dephasing time. Rephasing positive and negative beatmaps at 550 cm^{-1} showing the pattern expected for a two electronic level displaced harmonic oscillator comprising of GSB and SE vibrational coherence.[6] Beatmaps are shown as white-yellow-red heat maps and amplitude normalized to 1. Black dashes indicate the position of the 0-0 transition; thin dashes are drawn at ± 550 cm^{-1} from the 0-0 transition. DFT structure showing the displacement vectors associated with the ring breathing at 530 cm^{-1}

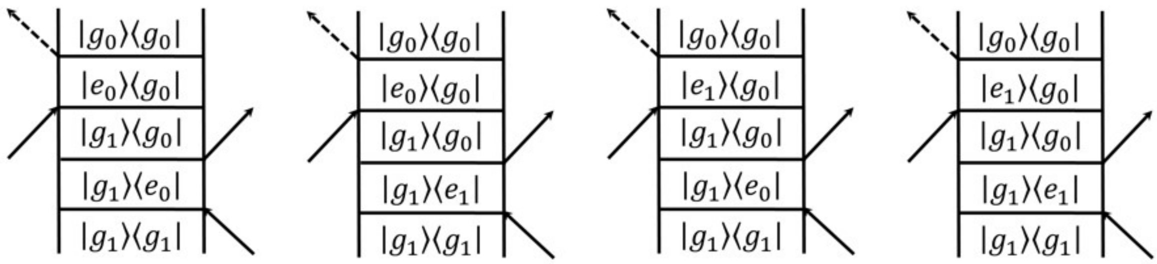

Figure S 9 Rephasing double-sided Feynman diagrams accounting for hot ground state bleach pathways oscillating at negative frequencies during *T*. g and e are the electronic ground and excited states and the subscripts indicate the number of quanta of a Raman active low frequency (below 200 cm^{-1}) molecular vibration coupled to the electronic transition.

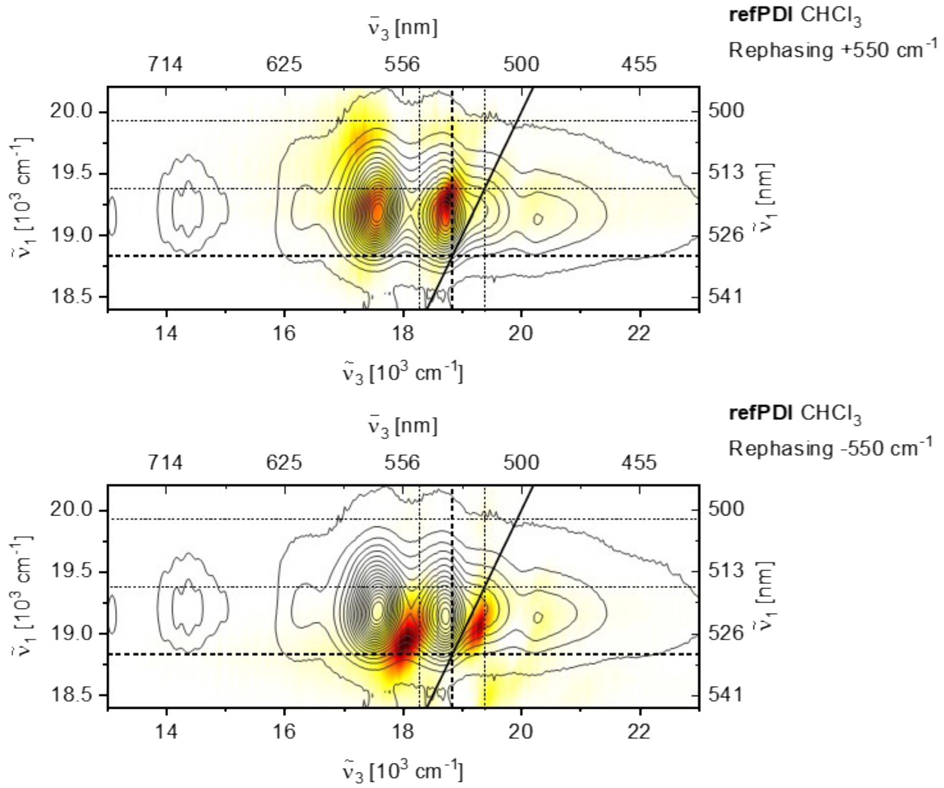

Figure S 10 Rephasing positive and negative beatmaps at 550 cm-1 of **refPDI** in $CHCl_3$ showing the pattern expected for a two electronic level displaced harmonic oscillator comprising of GSB and SE vibrational coherence. Beatmaps are shown as white-yellow-red heat maps and amplitude normalized to 1. Black dashes indicate the position of the 0-0 transition; thin dashes are drawn at ± 550 cm^{-1} from the 0-0 transition The redshifted signals at detection frequencies of~17500 (positive) and 18000 cm^{-1} (negative) are due to anharmonic coupling between the 550 cm^{-1} PDI breathing and the1350 cm^{-1} stretching mode giving rise to the strong vibronic progression evident in the absorption and emission spectra of **refPDI**. Anharmonic coupling between low frequency modes and the vinyl stretch was previously reported by us for other PDI derivatives in chlorinated solvents.[7]

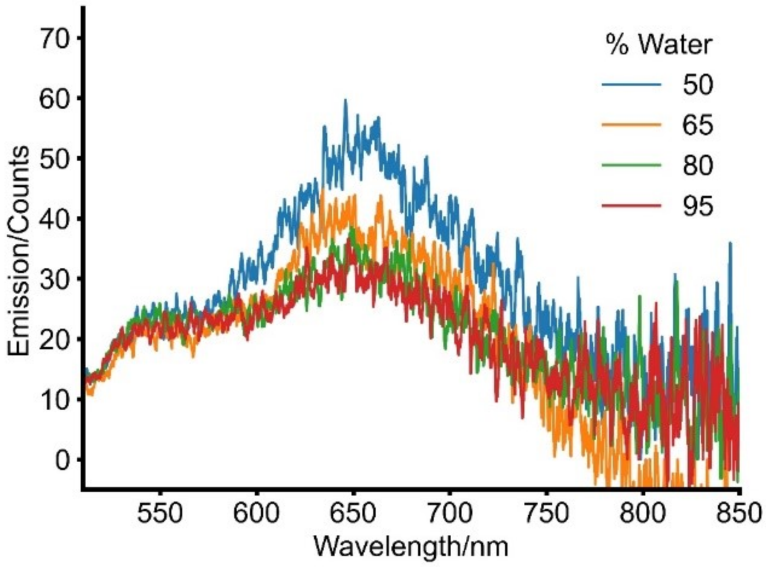

**Solvent ratio** (DMSO:H_2O)	**valPDI$_2$ FQY** (%)
50:50	6.5
35:65	4
20:80	5
5:95	4

Figure S 11 Emission spectra and FQY (table) of 1 µM solutions of **valPDI$_2$** in different DMSO/water volume fraction solvent mixtures. Data were measured on an Edinburgh Instruments FLS1000 spectrometer with 0.1 nm step size and 250 ms dwell time using an a PMT-980 photomultiplier tube and an integrating sphere (for FQY measurements). All measurements were taken with 3 nm excitation bandwidth and 0.95 nm emission bandwidth and samples excited at 493 nm.

Coordinates

valPDI2 in H_2O

O	14.31065	-2.84217	12.64182
O	11.52014	-7.55509	11.75193
O	17.19156	-4.50085	15.76230
O	13.98778	-8.98419	15.33146
O	16.07215	-6.77954	12.41802
O	14.98067	-9.67667	12.54505
N	15.82792	-3.62861	14.16436
O	12.66662	6.28916	19.11902
O	9.41787	2.12398	17.11630
N	12.68966	-8.32513	13.56519
O	15.99528	4.83191	21.87413
O	17.77983	-8.22625	12.79791
C	14.84400	-2.72031	13.73777
N	14.46131	5.64537	20.38974
C	16.00154	-2.54447	16.34494
C	14.54163	-1.60494	14.63993
C	11.21825	-2.26039	15.42577
C	13.58527	4.16833	18.64928
C	13.81281	1.80578	17.11757
C	14.90267	-0.38956	16.73867
N	10.54937	1.35501	18.94980
C	16.81878	-7.86382	12.15179
C	14.03290	0.63662	16.28025
C	14.45922	3.14727	19.06440
C	13.36402	-8.09327	14.76729
C	16.54950	-2.48306	17.61196
H	17.18939	-3.28266	17.95089
C	13.26507	-6.74390	15.34587
O	14.46086	-10.01852	10.36773
C	11.50292	-3.50246	14.72634

C	16.38512	-3.63184	15.44171
C	12.60263	-3.01906	17.33971
C	14.54233	1.93225	18.33056
C	12.89616	-4.25780	16.63557
C	12.80678	3.97876	17.52317
H	12.11933	4.75238	17.21936
C	10.99500	-3.77470	13.46030
H	10.40314	-3.04072	12.93997
C	16.31571	-1.38870	18.42967
H	16.77822	-1.37894	19.40233
C	11.92332	-5.99303	13.46736
C	15.53427	-0.31629	18.00901
C	13.70901	-5.24862	17.17891
H	14.19812	-5.09522	18.12615
C	12.02524	-7.31549	12.84447
C	10.41270	-1.26867	14.87386
H	10.01073	-1.38090	13.88114
C	12.58303	-9.68642	12.98049
H	12.36114	-9.48967	11.93877
C	13.70060	-0.59342	14.21638
H	13.24368	-0.66135	13.24192
C	16.29684	-8.57346	10.93338
H	17.09562	-9.17203	10.50284
H	15.96576	-7.86405	10.18037
C	15.39532	0.89661	18.79789
C	13.45761	0.50952	15.02003
H	12.81325	1.28113	14.63435
C	13.50313	5.43860	19.38802
C	15.23477	3.32675	20.22367
C	12.31130	-4.48400	15.36043
C	13.88157	-6.47466	16.55398

H	14.48457	-7.23686	17.02171
C	15.14647	-1.51685	15.90711
C	11.39404	-0.88836	17.45012
C	12.92908	2.82191	16.76562
H	12.32955	2.73919	15.87453
C	15.13902	-9.49871	11.22734
C	11.73679	-2.06805	16.73474
C	11.19811	-4.99764	12.84019
H	10.76681	-5.18758	11.87003
C	16.02903	2.29196	20.67938
H	16.61795	2.42983	21.57250
C	17.80330	-3.97912	12.71141
H	18.46848	-4.06214	13.57141
C	13.09155	-2.75852	18.61587
H	13.74889	-3.45878	19.10236
C	16.42464	-4.51195	13.13814
H	15.74911	-4.43067	12.29288
C	10.55689	0.07560	16.86203
C	12.49824	-5.74291	14.72496
C	10.09164	-0.11768	15.57478
H	9.45660	0.62869	15.12421
C	16.09509	1.09367	19.98459
H	16.74172	0.32395	20.37134
C	11.35878	-10.43827	13.53526
H	10.51737	-9.76730	13.35057
C	13.86928	-10.48963	12.98984
H	13.75402	-11.32794	12.31144
H	14.15900	-10.84212	13.96613
O	11.78109	0.61538	20.72139
O	12.40098	6.54763	22.05954
C	10.13558	1.25993	17.61060

C	11.10656	-11.70999	12.73216
H	11.12994	-11.52153	11.65959
H	10.12677	-12.11414	12.97812
H	11.84069	-12.48215	12.95651
C	15.27023	4.62405	20.90494
C	12.73569	-1.61447	19.31335
H	13.11183	-1.45252	20.31119
O	10.49694	4.52040	20.69193
C	11.87914	-0.68541	18.75488
C	16.41823	-5.97118	13.57501
H	17.37017	-6.29683	13.96881
H	15.65037	-6.15347	14.31798
C	9.88235	2.41443	19.74199
H	9.64523	3.17131	19.00242
C	17.73642	-2.51670	12.29265
H	17.06322	-2.38009	11.44701
H	18.72340	-2.17139	11.99191
H	17.40125	-1.86765	13.09900
C	14.61418	6.97895	21.02820
H	15.62483	6.95249	21.43061
C	11.39598	-10.73108	15.02734
H	12.23212	-11.37378	15.29517
H	11.46690	-9.82437	15.62166
H	10.47963	-11.24217	15.31858
C	11.42922	0.45841	19.55661
C	18.38283	-4.82253	11.58265
H	18.58362	-5.84467	11.88970
H	19.32774	-4.39545	11.25277
H	17.71167	-4.84767	10.72424
C	14.59476	8.16567	20.05430
H	13.58841	8.32513	19.68458

O	11.54127	8.58664	21.58305
C	13.73263	7.06180	22.26816
H	13.68033	8.07576	22.64543
H	14.13922	6.41695	23.03849
C	7.70836	3.03014	20.86838
H	8.12230	3.38413	21.80977
H	6.69779	2.68069	21.07047
H	7.63996	3.87572	20.18585
O	10.10433	4.67094	22.92162
C	8.53746	1.89472	20.28250
H	8.00548	1.52747	19.40281
C	10.79575	3.09881	20.74884
H	11.83690	3.01158	20.46786
H	10.67273	2.74413	21.76204
C	15.52248	7.90464	18.87432
H	16.53918	7.70474	19.21524
H	15.55654	8.77787	18.22595
H	15.20347	7.06204	18.26636
C	15.03958	9.42781	20.78629
H	14.34868	9.71796	21.57329
H	15.09268	10.25746	20.08407
H	16.02887	9.30386	21.22849
C	8.65375	0.74110	21.26632
H	9.13990	-0.12671	20.82788
H	7.65937	0.43111	21.58330
H	9.21057	1.02296	22.15801
C	11.41973	7.38949	21.72095
C	10.22519	5.17963	21.82547
C	10.10685	6.65532	21.55793
H	9.75219	6.84441	20.54852
H	9.39675	7.09307	22.25426

O	17.33779	-6.04092	18.12792
H	17.24892	-5.52079	17.31395
H	17.09888	-6.94918	17.88665
O	15.26510	-5.61936	19.88061
H	16.07750	-5.69291	19.34600
H	14.89567	-6.52033	19.90095
O	18.60287	3.81867	21.91097
H	18.61644	3.09042	22.54116
H	17.75284	4.27483	22.02816
O	19.13170	2.95412	19.38675
H	18.85539	3.29224	20.26344
H	20.08249	3.10499	19.35296
O	14.02753	0.42938	22.42520
H	13.37318	0.45427	21.71122
H	13.51627	0.10006	23.19683
O	13.55678	-6.92483	9.76862
H	13.23469	-6.52469	8.95248
H	12.76631	-7.18438	10.25895
O	12.11749	-1.41893	11.31612
H	12.82078	-1.97251	11.68636
H	12.24891	-1.42736	10.36417
O	19.38125	0.19205	18.89485
H	19.11093	1.09787	19.11765
H	19.11631	-0.37178	19.64730
O	10.85623	2.57433	13.83312
H	10.12449	2.78441	14.42272
H	10.97344	3.34070	13.26270
O	18.97320	-0.59431	16.35491
H	19.86640	-0.56801	15.99905
H	19.07335	-0.35466	17.30452
O	17.13989	1.27265	15.55890

H	17.77306	0.57936	15.83350
H	17.42048	2.07909	16.02662
O	10.92568	2.28802	24.17838
H	10.53662	3.12335	23.87643
H	10.86350	2.29918	25.13810
O	16.36266	-8.57717	17.15251
H	17.19729	-8.68366	16.65152
H	15.66639	-8.90391	16.56595
O	16.31835	0.55660	13.01279
H	16.44450	1.31495	12.43463
H	16.59746	0.85030	13.89956
O	9.07107	-2.63662	19.75985
H	8.22086	-2.92075	20.11223
H	8.92385	-2.53882	18.79647
O	18.60038	-8.58998	15.56285
H	18.99489	-7.69528	15.51233
H	18.40420	-8.81317	14.64539
O	16.14449	-1.37856	22.20584
H	15.63298	-2.15759	21.90749
H	15.48609	-0.67273	22.32276
O	10.90782	6.63062	16.92022
H	10.92164	7.52838	16.57677
H	11.41949	6.64031	17.74451
O	8.53663	-2.59463	17.08567
H	8.56839	-3.42903	16.58336
H	7.97684	-1.99684	16.55779
O	18.04944	3.53013	16.94603
H	18.83275	3.89211	16.51779
H	18.32741	3.30319	17.85612
O	19.52507	-6.02098	15.22218
H	18.81670	-5.43444	15.52701

H	20.34781	-5.70811	15.61127
O	6.75766	1.56018	16.64818
H	7.66917	1.85457	16.79898
H	6.30531	2.30468	16.23984
O	15.79954	5.17527	16.73297
H	15.86082	5.44846	15.81216
H	16.54522	4.56157	16.86451
O	8.71351	4.92316	16.92023
H	9.05211	4.02126	17.01356
H	9.49266	5.50146	16.83051
O	13.39938	3.09288	22.77630
H	13.81534	2.20970	22.69287
H	12.62986	2.92783	23.34151
O	6.88595	-0.93094	15.55572
H	5.97320	-1.23463	15.56277
H	6.86577	-0.03972	15.95885
O	9.95710	0.34531	12.23418
H	10.35339	1.05004	12.77343
H	10.69685	-0.18774	11.90946
O	12.10517	-0.36103	24.16812
H	11.60422	-1.02554	23.67248
H	11.61092	0.46461	24.06727
O	14.59174	-3.50002	21.46612
H	14.24895	-3.91793	22.25891
H	14.93059	-4.23162	20.91707
O	8.41887	-4.92363	15.55765
H	8.36201	-4.57156	14.64385
H	7.59055	-5.39021	15.70927
O	7.70443	-1.23575	12.89128
H	7.49917	-1.00197	13.81499
H	8.40348	-0.62411	12.59991

O	18.54615	-1.46885	20.92328
H	17.75463	-1.41361	21.49537
H	18.54582	-2.37528	20.57875
O	8.00341	-3.92336	13.05812
H	7.93285	-2.94412	12.99679
H	7.29469	-4.26114	12.50538
O	10.35194	-6.42053	17.01355
H	9.75853	-5.94316	16.41285
H	10.41698	-5.85263	17.80603
O	14.40474	-8.21445	19.38852
H	15.11847	-8.51785	18.81124
H	13.58577	-8.31325	18.87168
O	10.84713	-1.74681	22.03307
H	10.30424	-2.14974	21.34380
H	11.22997	-0.95116	21.63541
O	10.57074	-4.93725	19.33347
H	10.10006	-4.09452	19.47289
H	10.57079	-5.38478	20.18382
O	7.83532	6.00182	19.27920
H	8.10740	5.63093	18.41363
H	7.09095	6.57801	19.08609
O	11.94886	-8.52876	17.95709
H	11.45268	-7.77477	17.59884
H	11.35899	-8.95418	18.58766
O	18.64737	-4.01412	19.65122
H	18.22938	-4.82033	19.31453
H	19.57437	-4.07672	19.41304

valPDI2 in chloroform

O	9.84407721486109	1.68155878311809	14.59605779719188
O	2.28992380300576	3.88583746602193	16.29683509096175

O	10.77072901729168	-0.92852466069473	18.18195473545020
O	3.21804442447638	0.63150735295098	19.29097668594321
O	8.17496566429387	-1.42664074695548	15.23125724423013
O	4.27512664267757	0.41674161366244	15.13807345281385
N	10.24487679908361	0.35767381428421	16.39019718735362
O	7.31007855466894	10.19747574405079	21.57823245288597
O	0.90363099168139	11.23611394289221	24.74129911304726
N	2.59179014806421	2.22313011046789	17.80560288940572
O	9.35289479898571	7.99524041767818	24.96645746259547
O	7.03184670174604	-2.57687363232062	16.77684712479707
C	9.89053497931438	1.56062682440727	15.80944612042972
N	8.43533677789366	9.14314425949662	23.24014988268774
C	10.24898774524218	1.32286662640365	18.62404273267774
C	9.58517677237363	2.67556620631258	16.71321086009005
C	1.53226727863105	7.26553622572503	21.74156309399483
C	8.19335737751400	8.08247394099936	21.06979168805839
C	8.69308353276836	5.92935917044781	19.32336893785668
C	9.55608808353863	3.64401214417944	18.96303150053677
N	1.02036007366332	9.55123100027459	26.26161223953412
C	7.06690204086101	-1.85127487493386	15.81548047967480
C	9.01880450126582	4.84130549528716	18.42240736072834
C	8.88168396788999	6.94925083705079	21.54094827117563
C	2.83892506184930	1.77600520833628	19.09545062143941
C	10.51946686074448	1.21278162800374	19.97470845979504
H	10.86619978763864	0.26618516539693	20.36022928272152
C	2.60547954990308	2.71769862764735	20.18476384959859
O	5.69277353353916	0.34095477828395	16.87602216630160
C	1.77923832895870	6.33221863523700	20.66011294895712
C	10.44276551536055	0.15770158686765	17.75098278925050
C	1.83899139048033	5.43225640272608	23.38973832020940
C	9.11499876482303	5.84772814333012	20.67662750158317

C	2.09542507615738	4.50047270238173	22.30681491835714
C	7.74654807286117	8.11440942240680	19.75931109330501
H	7.21346176660318	8.98330532460675	19.40806847765538
C	1.77549536127392	6.73003169065324	19.32840297957738
H	1.59447099167981	7.75512349445301	19.06068389315777
C	10.36485803328910	2.30709620490899	20.81242990386938
H	10.61523705680030	2.18708738991037	21.85228174935441
C	2.23069142456700	4.49422849657978	18.56997146421152
C	9.89482377505505	3.52559562485506	20.33660576884617
C	2.40819408050253	3.16483529171264	22.53702265634630
H	2.46918333394929	2.78073648997732	23.54115970487059
C	2.37160676013585	3.54112754664367	17.47052543381811
C	1.23030371134674	8.60249108319044	21.51319157588437
H	1.14377200565660	8.97860767872804	20.50921855911733
C	2.61577005835748	1.21443855576546	16.74277169256102
H	2.95737021422118	0.29230773341879	17.22503846741743
C	9.14820328443497	3.88318672419563	16.20055801376349
H	9.04109351378246	3.99697713310333	15.13294872821670
C	5.84103395853695	-1.31057416425035	15.10870211233220
H	6.08926554324781	-1.02030525050173	14.08746714945298
H	5.05957792970898	-2.07244773811622	15.09035907375679
C	9.75683954788568	4.68928143091406	21.19140445978579
C	8.84481611240230	4.93964863416496	17.04703382891761
H	8.49326253157854	5.85632328662367	16.60417208106131
C	7.94257894131457	9.21882476785498	21.95051175836311
C	9.34056098790311	6.91993602583716	22.87295922997392
C	2.05230267796256	4.97310582441866	20.96793238527572
C	2.67146493224858	2.28765927749675	21.49838538232425
H	2.93355207587314	1.26365185234784	21.71049255071245
C	9.78534814183502	2.54370161194816	18.10066927402221
C	1.38598415703688	7.71576062287766	24.13616634469383

C	7.99183568091953	7.05506155526533	18.90393743509393
H	7.61549354383844	7.12024534629409	17.89793720651284
C	5.28243953370701	-0.09522073964651	15.83257310821547
C	1.59232657246522	6.79581372579094	23.07966049988827
C	1.98925294113176	5.83008557206220	18.30091974444896
H	1.95668639702678	6.16887115532295	17.27975814703907
C	10.05219030550660	5.81719680603383	23.31636383876898
H	10.46843880917899	5.81655088981442	24.31028152217190
C	11.92060249135372	-1.29902806000180	15.54165877582722
H	12.03921888555027	-1.91918382226059	16.43385486292172
C	1.80684745464980	5.03238277340748	24.72143274292296
H	1.95034436018151	3.99869990653961	24.98762351967880
C	10.47143488323235	-0.77590263394393	15.48724537464365
H	10.28226878883003	-0.38105413812976	14.48236017524768
C	1.15050241153882	9.07555188022828	23.86651914567502
C	2.29218214712466	4.06186099956542	19.90836281747206
C	1.05111924427231	9.50043906799407	22.55495073054072
H	0.84193890507739	10.53977649024005	22.35064357920944
C	10.23529455689931	4.71697090928798	22.49639803923158
H	10.76947657663070	3.87552793346377	22.90093501968310
C	1.20209333577680	0.96896110489794	16.18921679354222
H	0.82875742115450	1.90524726347593	15.76198930534797
C	3.64753060332720	1.58061415935869	15.66890439059088
H	3.19053305856273	2.09045412722421	14.82165041199420
H	4.41596466096638	2.21956391612952	16.11257640587033
O	1.20906728335147	7.82559771296365	27.73441163926519
O	6.44787265542719	10.86517221995218	25.67533507277808
C	1.01641692165847	10.04549994621033	24.95754680727164
C	0.27109818874308	0.51674802916537	17.31279105971160
H	-0.73486946682798	0.37189421768750	16.92775214508464
H	0.22273258525793	1.24884848527130	18.11486723579917

H	0.61860161485271	-0.42657168776125	17.72926405179233
C	9.06400557346906	8.03330534683700	23.77445787781626
C	1.57732470452920	5.93568909497777	25.74655689145607
H	1.54107930279124	5.60018472602413	26.77187480399171
O	3.11970641947214	10.45842257408179	28.07887478883741
C	1.39469347858120	7.27852944640335	25.47105459158650
C	9.43099607687530	-1.86174065986687	15.76347232918057
H	9.70732815005521	-2.78961777935010	15.25660041531829
H	9.33025616073549	-2.06458082560084	16.83265888642201
C	0.81243611005079	10.57511281726613	27.29962446690981
H	1.08217554573648	11.51468912676696	26.80106166741029
C	12.90430831462547	-0.13050970855100	15.61371535873182
H	12.70532843493353	0.58588879011645	14.81883728773161
H	13.92130710577604	-0.49695101712100	15.50082817148686
H	12.84128532332840	0.38451738997560	16.56956201951702
C	8.19119054028843	10.27808251773615	24.13824905927963
H	8.60710362126576	9.98937852508219	25.10784706721273
C	1.24358638088009	-0.10383536821699	15.10449854731020
H	1.62769269926893	-1.03460551327555	15.51682550696529
H	1.87494810709195	0.18975144026908	14.27159673908698
H	0.23963517460504	-0.28202120664818	14.72793194876813
C	1.19448924680066	8.22333542670572	26.57908474495207
C	12.22012877231775	-2.13462585970447	14.29888117105312
H	11.55425800068725	-2.98861105092214	14.21333513031220
H	13.23911580100449	-2.51025261983167	14.34609948549660
H	12.12058543397153	-1.52742653169994	13.40165729756121
C	8.92469031152754	11.54468776378750	23.66532827702958
H	8.58944062736961	11.78983259443410	22.65495005312257
O	4.23903393892428	10.88467887643795	25.30585220450492
C	6.68396223197055	10.45898321601280	24.32762408714794
H	6.27357204214067	11.20205358441543	23.63897337567955

H	6.16679802809991	9.50766946401973	24.15556966741226
C	-1.02721112802762	12.10761543817680	28.07397789111601
H	-0.45701727357521	12.42334627209323	28.94400994310820
H	-2.08525275762951	12.18187073014967	28.31324148276821
H	-0.81108811885812	12.78897890556928	27.25502710535811
O	3.12431271804770	12.70163310442726	28.15480452169764
C	-0.68055012176406	10.67171339197739	27.68437458590069
H	-1.24691041748879	10.42534825511259	26.77822383379854
C	1.75320024472888	10.42981878684878	28.49516648908774
H	1.57154224304279	11.27074400313098	29.17126631410393
H	1.62318199289977	9.48264483104412	29.01434226525594
C	10.42986180225485	11.29030205589259	23.64737421197270
H	10.79939471108759	11.12990838604029	24.65796561966417
H	10.94780403613562	12.14712090269084	23.22513814025850
H	10.67547557615019	10.41610584958096	23.04972450583591
C	8.60811592160267	12.72112637515015	24.58313544483694
H	7.55963303182539	12.99801675555586	24.52888294534486
H	9.19793898569986	13.58269017091811	24.28124253539101
H	8.85269923512040	12.47770060506783	25.61394940643942
C	-1.10005007218433	9.70331681810339	28.78805401381863
H	-0.77356950480378	8.69096347008720	28.57110512771416
H	-2.18415024896432	9.70260914371249	28.87706876940645
H	-0.68255375445441	10.00570046666898	29.74506406728758
C	5.18418676787028	11.05568015982034	26.03193335959769
C	3.67627618694041	11.66055585284755	27.93289459464718
C	5.11515734080968	11.52695144280291	27.47186374707356
H	5.61106648577299	12.49607798221363	27.54495850286192
H	5.64438946718497	10.80642673127680	28.09745014812352
Cl	6.61593055019986	2.33703452855161	19.46097408196550
C	6.22319560803026	0.65356325755048	19.80527576210054
Cl	7.59438084031592	-0.38095251750455	19.40726006359499

Cl	5.79716083419214	0.49711307271952	21.50194717532228
H	5.36542489367643	0.36402234194457	19.19340758930587
Cl	5.12227787757512	6.68543300813381	22.71597613155056
C	5.84303446241287	5.35400648431323	21.80236996168723
Cl	5.41151616005671	3.81078894236874	22.52710828106200
Cl	5.31154954581973	5.40109175054551	20.12341974134283
H	6.92893545974351	5.45844111406414	21.83148224398336
Cl	4.07638421174689	9.79095594146571	22.39537021736549
C	4.38098620515758	10.05647851447094	20.67489609409760
Cl	4.40236599604286	8.50045743410389	19.83526704138773
Cl	3.14323699192142	11.09042562328686	19.97427739985861
H	5.35910078627995	10.53092841818830	20.56840665747919
Cl	4.80496525713668	6.97258279302859	17.14739451804021
C	5.02078441097306	5.53641668250000	16.14612089138090
Cl	6.10117013016374	5.89575470592610	14.80113283938241
Cl	5.68589352425494	4.23302632809294	17.14959139254063
H	4.04707702156160	5.21976943602267	15.76056902456877
Cl	6.68067360078053	2.37794927734015	14.55501387008719
C	7.44393349123588	2.23943834959368	12.96731584575492
Cl	6.74597269943231	0.87856576487982	12.08685610597528
Cl	7.21735538770719	3.73783570481644	12.05516664510780
H	8.51183754161623	2.05788615853126	13.12565249781611
Cl	1.76028077233767	13.25320701929966	22.26825703467326
C	3.03748145099958	12.99473269652610	23.45079604243192
Cl	4.60967109923176	13.12244245313583	22.65979680834839
Cl	2.92462529818316	14.17802162764739	24.75628761941099
H	2.93703021750727	11.99441500565561	23.87726654544625
Cl	4.50058987425222	7.80864990502824	25.84211942287805
C	4.32564780294034	7.20857073274442	27.50083704321363
Cl	4.43813231072046	5.45045759219499	27.51144504641094
Cl	5.57918538094739	7.91159688332309	28.51737541880120

H	3.34633082390587	7.50222252168275	27.88994951685238
Cl	-0.46812902851282	6.86656449034675	15.88503020850373
C	0.79952240979234	6.20585193694766	14.84425830218483
Cl	0.17616607600717	5.96088033290287	13.21062416101668
Cl	2.17894912673529	7.32490745336658	14.80651640256744
H	1.14004696298642	5.24690828171328	15.25257291004724
Cl	7.11115696841538	5.34738186374972	25.09536959680037
C	8.40282202544659	5.36009126605069	26.30710901891637
Cl	7.72088836645051	5.36997859206606	27.93093526571260
Cl	9.43696511431790	3.94570221365313	26.09950548383390
H	8.99121629540888	6.27009622412155	26.14933504624554
Cl	12.27579695855001	5.54629816127416	19.01754218934334
C	13.00850681916274	4.72204320145257	17.63707514627830
Cl	12.99092206379779	2.97112172597170	17.89256549609137
Cl	12.14056181233791	5.13138944755081	16.15469808298322
H	14.04559221001788	5.04870061554305	17.54342523598471
Cl	11.75482951606777	8.11595918788823	20.81340357000151
C	11.92064514746139	9.74682826323204	20.15092668315573
Cl	10.33046355516212	10.51305476440559	20.07269978462309
Cl	13.01914405069373	10.70328939003370	21.14815433597048
H	12.32179086264631	9.67466457451499	19.13852790757149
Cl	8.05738568714224	2.41937485277392	23.38655772742014
C	8.85031891141963	0.97009290970843	24.00264887961405
Cl	8.83232813008891	-0.30065533097775	22.77184653753763
Cl	10.51571038976363	1.34421237590251	24.47197489531667
H	8.30848591401775	0.62901328206807	24.88688704492594
Cl	-1.90516602904895	7.89976347321312	22.17672262357610
C	-2.48986507722188	8.61663788125817	23.68316166427738
Cl	-1.81896765836483	7.74013841929060	25.06877317731321
Cl	-2.03651898162262	10.32647602839944	23.76723824826411
H	-3.57729991222606	8.53200653763233	23.71350631202443

Cl	13.17757444656262	9.10737986600648	26.74823404573479
C	12.30991990094370	8.10761279244713	25.58066918149351
Cl	12.77428137932001	8.54868449875582	23.93516920094385
Cl	12.65904575370570	6.39720675568826	25.86732910740666
H	11.23218450204580	8.26553574731944	25.68663889040706
Cl	-0.47732734363946	2.09584391526949	23.56039657627996
C	-1.82169150299821	3.23772190743942	23.67605185717000
Cl	-1.29409436055440	4.85671311628692	23.20297977251067
Cl	-3.15782494312111	2.71562641874091	22.64351273735283
H	-2.17071191651627	3.27260943099268	24.70950538716658
Cl	1.12752757187980	-2.85634609989817	20.45647331157916
C	1.62173360760642	-1.24684332702752	20.97956470235812
Cl	0.22273745178163	-0.16443601253151	21.02757320445392
Cl	2.37060840400854	-1.32436375509388	22.58233221534899
H	2.34752699682658	-0.83465516975088	20.26993813502294
Cl	6.03559982696595	10.21633784866000	17.39509358042191
C	4.44893870650057	10.24920063359712	16.63903651066229
Cl	3.81824527375947	11.89857354810936	16.63835051506573
Cl	4.55962787503325	9.62009622432870	14.99223453833674
H	3.78147033481347	9.60997518674770	17.22025077190210
Cl	13.18298607893058	3.44802063334574	24.07862360016684
C	13.89847449175009	4.10788158024122	22.59929131655983
Cl	13.50404118949640	3.07926571319002	21.21351328085359
Cl	13.31117183938368	5.75009378121002	22.30908955140120
H	14.98292356960306	4.13564044538492	22.71699408131615
Cl	-1.37678689245007	5.68862808833851	19.90269322878435
C	-1.53079115093285	4.04296526122007	19.27328280948696
Cl	-0.87474879543657	3.95850052380551	17.63459950043430
Cl	-0.68213060845775	2.90360293532615	20.32539671324159
H	-2.58791918785083	3.77300546986228	19.24456836620345
Cl	4.95246692216806	1.24372716905738	24.43402090823974

C	5.03016665260727	2.48594387346537	25.70015854284425
Cl	3.60512295723259	2.38275098154141	26.73838025163129
Cl	6.49390456462881	2.28360276202927	26.65745786322690
H	5.05710645049257	3.47131165561707	25.22976767943605
Cl	4.20594127139493	-4.83120273126832	18.80802232761854
C	4.63595370495074	-3.12246486350169	18.64762559939433
Cl	3.40031198161514	-2.28868344549917	17.70934491335810
Cl	4.77704956109589	-2.37887591243511	20.24363376702584
H	5.59096986119444	-3.03128901422726	18.11649088389615
Cl	10.64641505726709	8.05846711731438	17.49520784428847
C	9.54640602279028	9.26653792206457	16.81361022359573
Cl	10.47216158133985	10.61529129359802	16.14830836178059
Cl	8.53951937348855	8.52607874752981	15.56639196714682
H	8.90314606020000	9.64227167896487	17.61252949549729
Cl	8.27719525128219	13.97922247554444	20.66868664149625
C	7.51553680719007	12.67080524784129	19.75913718345380
Cl	5.83050412992797	13.07114859335827	19.40443562143141
Cl	8.39572034806290	12.39208293195557	18.25269262168306
H	7.54421904164318	11.75698015751349	20.36054491108152
Cl	-0.14656230048438	11.33270760042838	19.41810146251213
C	-0.45925806588074	10.16305158283805	18.12031264174207
Cl	1.04551193610644	9.74496355519130	17.30078663336967
Cl	-1.22045457073584	8.70137764427124	18.77617696869504
H	-1.13472648477534	10.61217236229507	17.39036035752153
Cl	10.00494936010599	10.39275482491929	27.41070747690094
C	9.09943201518092	9.00002438370663	28.00217429039092
Cl	10.21026987248677	7.66378418784330	28.34060771060911
Cl	8.20934794945573	9.44043224310871	29.46570321312153
H	8.39169236379437	8.68384322252965	27.23319145863710

References

(1) Hestand, N. J.; Spano, F. C. Interference between Coulombic and CT-Mediated Couplings in Molecular Aggregates: H- to J-Aggregate Transformation in Perylene-Based π-Stacks. *J Chem Phys* **2015**, *143* (24), 244707. https://doi.org/10.1063/1.4938012.

(2) Hestand, N. J.; Spano, F. C. Expanded Theory of H- and J-Molecular Aggregates: The Effects of Vibronic Coupling and Intermolecular Charge Transfer. *Chem Rev* **2018**, *118* (15), 7069–7163. https://doi.org/10.1021/acs.chemrev.7b00581.

(3) Kim, T.; Lin, C.; Schultz, J. D.; Young, R. M.; Wasielewski, M. R. π-Stacking-Dependent Vibronic Couplings Drive Excited-State Dynamics in Perylenediimide Assemblies. *J Am Chem Soc* **2022**, *144* (25), 11386–11396. https://doi.org/10.1021/jacs.2c03993.

(4) Snellenburg, J. J.; Laptenok, S. P.; Seger, R.; Mullen, K. M.; Stokkum, I. H. M. van. Glotaran : A Java -Based Graphical User Interface for the R Package TIMP. *J Stat Softw* **2012**, *49* (3), 1–22. https://doi.org/10.18637/jss.v049.i03.

(5) Bressan, G.; Heisler, I. A.; Greetham, G. M.; Edmeades, A.; Meech, S. R. Half-Broadband Two-Dimensional Electronic Spectroscopy with Active Noise Reduction. *Opt Express* **2023**, *31* (25), 42687. https://doi.org/10.1364/OE.500017.

(6) Green, D.; Bressan, G.; Heisler, I. A.; Meech, S. R.; Jones, G. A. Vibrational Coherences in Half-Broadband 2D Electronic Spectroscopy: Spectral Filtering to Identify Excited State Displacements. *J Chem Phys* **2024**, *160* (23), 1–12. https://doi.org/10.1063/5.0214023.

(7) Bressan, G.; Penty, S. E.; Green, D.; Heisler, I. A.; Jones, G. A.; Barendt, T. A.; Meech, S. R. Ultrafast and Coherent Dynamics in a Solvent Switchable "Pink Box"

Perylene Diimide Dimer. *Angewandte Chemie International Edition* **2024**, *63* (39). https://doi.org/10.1002/anie.202407242.

(8) Hartmann, D.; Penty, S. E.; Zwijnenburg, M. A.; Pal, R.; Barendt, T. A. A Bis-Perylene Diimide Macrocycle Chiroptical Switch. *Angewandte Chemie International Edition* **2025**, *64* (15). https://doi.org/10.1002/anie.202501122.

(9) Hall, C. R.; Conyard, J.; Heisler, I. A.; Jones, G.; Frost, J.; Browne, W. R.; Feringa, B. L.; Meech, S. R. Ultrafast Dynamics in Light-Driven Molecular Rotary Motors Probed by Femtosecond Stimulated Raman Spectroscopy. *J Am Chem Soc* **2017**, *139* (21), 7408–7414. https://doi.org/10.1021/jacs.7b03599.

(10) Yan, S.; Tan, H. S. Phase Cycling Schemes for Two-Dimensional Optical Spectroscopy with a Pump-Probe Beam Geometry. *Chem Phys* **2009**, *360* (1–3), 110–115. https://doi.org/10.1016/j.chemphys.2009.04.019.

(11) Feng, Y.; Vinogradov, I.; Ge, N.-H. General Noise Suppression Scheme with Reference Detection in Heterodyne Nonlinear Spectroscopy. *Opt Express* **2017**, *25* (21), 26262. https://doi.org/10.1364/OE.25.026262.

(12) Bressan, G.; Green, D.; Jones, G. A.; Heisler, I. A.; Meech, S. R. Two-Dimensional Electronic Spectroscopy Resolves Relative Excited-State Displacements. *J Phys Chem Lett* **2024**, *15* (10), 2876–2884. https://doi.org/10.1021/acs.jpclett.3c03420.

(13) de A. Camargo, F. V.; Grimmelsmann, L.; Anderson, H. L.; Meech, S. R.; Heisler, I. A. Resolving Vibrational from Electronic Coherences in Two-Dimensional Electronic Spectroscopy: The Role of the Laser Spectrum. *Phys Rev Lett* **2017**, *118* (3), 033001. https://doi.org/10.1103/PhysRevLett.118.033001.

(14) Neese, F.; Wennmohs, F.; Becker, U.; Riplinger, C. The ORCA Quantum Chemistry Program Package. *J Chem Phys* **2020**, *152* (22). https://doi.org/10.1063/5.0004608.

(15) Bannwarth, C.; Ehlert, S.; Grimme, S. GFN2-XTB—An Accurate and Broadly Parametrized Self-Consistent Tight-Binding Quantum Chemical Method with Multipole Electrostatics and Density-Dependent Dispersion Contributions. *J Chem Theory Comput* **2019**, *15* (3), 1652–1671. https://doi.org/10.1021/acs.jctc.8b01176.

(16) Bussi, G.; Donadio, D.; Parrinello, M. Canonical Sampling through Velocity Rescaling. *J Chem Phys* **2007**, *126* (1). https://doi.org/10.1063/1.2408420.
